# Comparative proteomic analysis of nuclear and cytoplasmic compartments in human cardiac progenitor cells

**DOI:** 10.1038/s41598-021-03956-8

**Published:** 2022-01-07

**Authors:** Guillermo Albericio, Susana Aguilar, Jose Luis Torán, Rosa Yañez, Juan Antonio López, Jesús Vázquez, Carmen Mora, Antonio Bernad

**Affiliations:** 1 Department of Immunology and Oncology, Centro Nacional de Biotecnología (CNB-CSIC), C/ Darwin 3, Campus Universidad Autónoma de Madrid, 28049 Madrid, Spain; 2 Veterinary Faculty, Universidad Complutense de Madrid, Avda. Puerta de Hierro, s/n. Ciudad Universitaria, 28040 Madrid, Spain; 3 Hematopoietic Innovative Therapies Division, Centro de Investigaciones Energéticas, Medioambientales y Tecnológicas, Madrid, Spain; 4 Centro de Investigación Biomédica en Red de Enfermedades Raras, Av Complutense, 40, 28040 Madrid, Spain; 5 Instituto de Investigaciones Sanitarias de la Fundación Jiménez Díaz, Madrid, Spain; 6 Laboratory of Cardiovascular Proteomics, Centro Nacional de Investigaciones Cardiovasculares (CNIC), Melchor Fernández Almagro 3, 28029 Madrid, Spain; 7 CIBER de Enfermedades Cardiovasculares (CIBERCV), Madrid, Spain

**Keywords:** Cell biology, Cardiology

## Abstract

Clinical trials evaluating cardiac progenitor cells (CPC) demonstrated feasibility and safety, but no clear functional benefits. Therefore a deeper understanding of CPC biology is warranted to inform strategies capable to enhance their therapeutic potential. Here we have defined, using a label-free proteomic approach, the differential cytoplasmic and nuclear compartments of human CPC (hCPC). Global analysis of cytoplasmic repertoire in hCPC suggested an important hypoxia response capacity and active collagen metabolism. In addition, comparative analysis of the nuclear protein compartment identified a significant regulation of a small number of proteins in hCPC *versus* human mesenchymal stem cells (hMSC). Two proteins significantly upregulated in the hCPC nuclear compartment, IL1A and IMP3, showed also a parallel increase in mRNA expression in hCPC *versus* hMSC, and were studied further. IL1A, subjected to an important post-transcriptional regulation, was demonstrated to act as a dual-function cytokine with a plausible role in apoptosis regulation. The knockdown of the mRNA binding protein (IMP3) did not negatively impact hCPC viability, but reduced their proliferation and migration capacity. Analysis of a panel of putative candidate genes identified *HMGA2* and *PTPRF* as IMP3 targets in hCPC. Therefore, they are potentially involved in hCPC proliferation/migration regulation.

## Introduction

The adult mouse heart has low but intrinsic cardiomyocyte turnover^1–3^, mainly associated with the function of multipotent resident cardiac stem or progenitor cells (CSC/CPC) populations^4,5^. The eventual contribution of adult de-differentiating cardiomyocytes to heart homeostasis remains to be consolidated^6^. Several markers have been proposed to identify and purify CSC/CPC (reviewed in^4^) but the first^5^ and the more intensively studied has been the expression of c-Kit^+^ by CSC (reviewed in^7^), seeking to explode their expected potential for cardiovascular therapy^8–12^. In spite of the recent controversy^13–15^ several important cues have been provided during the last years, and main technical limitations at the origin of the conflict have been mostly clarified^16–18^. Results using these mouse lines (c-Kit-Cre/Rosa26-floxed-STOP-reporters)^14,15^ or dual-recombinase approaches^13^, concluded that cardiac c-Kit^+^ populations have a marginal cardiomyogenic capacity (0.01% of total cardiomyocytes). Torella et al. provided strong evidences indicating that Kit^Cre^ alleles, both inducible and constitutive, promote an inefficient recombination of the several reporter constructs evaluated in the cardiac c-Kit^+^ cells, including a very low recombination (≤ 1%) of c-Kit^+low^CD45-CD31-cells. Compelling experiments concluded that c-kit haploinsufficiency in all these genetic fate-mapping experiments is at the most probable origin of all discrepancies, provoking growth and clonogenesis, cardiosphere formation defects. All these deficits, including the cardiomyogenic differentiation capacity were completely rescued by BAC transfection, that harbors the complete cloned c-Kit locus, and, finally, Vicinanza et al. confirmed the described phenotypes in vivo, using cloned cardiac c-kit^+low^CD45^-^CD31^-^ cells^17^. Therefore, currently murine CSC/CPC population is defined as Sca‐1^pos^/c‐kit^pos^/CD31^neg^/CD45^neg^/Tryptase^neg^ ^10,18^, distinguishing them from cardiac c‐kit^pos^ endothelial (CD31^pos^) and mast (CD45^pos^/Tryptase^pos^) cells; CSC/CPCs are a rare population in the adult heart^19^. The recent identification of c-Kit^+^ stem cells in adult vessels^20^ and the characterization of human cardiac atrial myxomas as the first-described CSC (c-Kit^+^CD45-CD31-)-related human heart disease^21^, clearly offer indirect support for ckit^+^ CSC.

Cardiosphere-derived cells (CDC) and c-kit^pos^ CPC have been characterized and evaluated in pigs and humans. Results from transplantation studies in swine models of cardiac ischemic injury revealed a moderate but reproducible improvement in cardiac function^22–27^. Multiple lines of evidence from preclinical studies on the transplantation of human or swine CSC/CPC suggested that the mechanisms of action are mainly indirect (reviewed in^28^), resulting in durable benefits despite low engraftment and cell survival of the transplanted cells^29,30^.

Human c-kit^pos^ CPC (hCPC) showed weak expression of classical embryonic pluripotency factors (*OCT4, NANOG, SOX2* and *ECAD*). These data and cell membrane expression analyses, coupled with their demonstrated immunoregulatory capacity, indicated that hCPC could be a resident mesechymal stem cell (MSC)-like population^31–35^. Finally, analysis of the hCPC secretome revealed a strong angiogenic potential and highlighted CXCL6 as an important paracrine factor that signals mainly through CXCR2^36^.

Based on promising preclinical results^22,24^, a phase I/IIa clinical trial was carried out, using allogeneic hCPC, for the treatment of pacients with large cardiac infarcts (EudraCT 2013–001,358-81;^37^). While the results demonstrated the feasibility and safety of the approach, no statistical significant functional benefits were demonstrated^38^. Given all of these data, a deeper understanding of hCPC biology and their behavior in response to acute or diffuse chronic damage might be critical for a better definition of the mechanism of action of these therapies, which might lead to improvements in the current strategies based on hCPC.

With this in mind, here have compared, by a proteomics approach, the differential cytoplasmic and nuclear compartments of hCPC, hMSC and fibroblasts. From this analysis, we focused on two overexpressed nuclear proteins in hCPC, IL1A and IMP3 (IGF2BP3). IL1A was demonstrated to be a dual-function cytokine with a plausible role in apoptosis regulation, and IMP3 regulated proliferation and migration of hCPC.

## Results

### Comparative analysis of the human CPC cytoplasmic compartment

**Figure 1:**
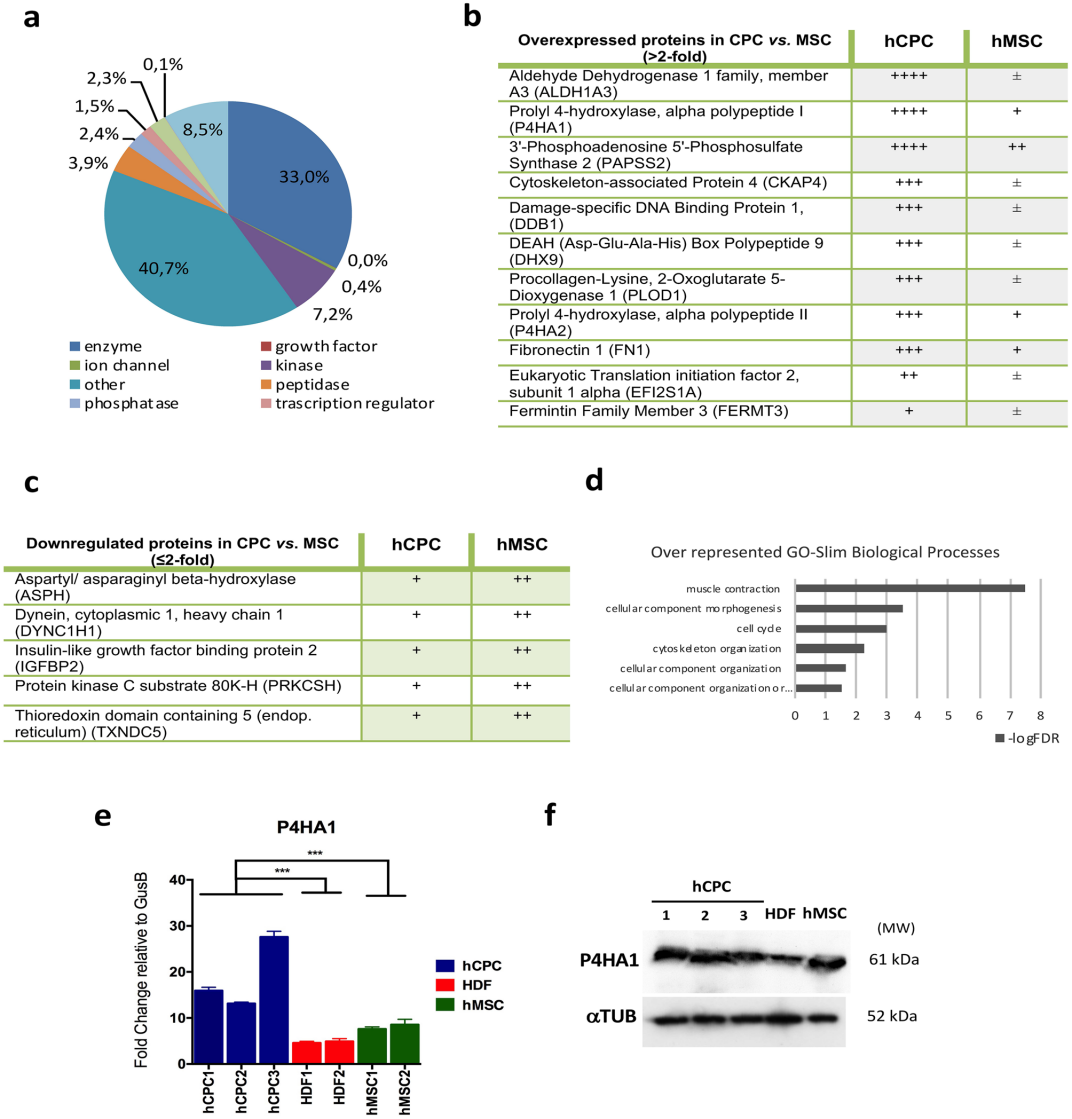
Comparative analysis of hCPC cytoplasmic compartment. (**a**) Ingenuity pathway analysis of the cytoplasmic hCPC subproteome obtained by whole label-free (LF) proteomics; (**b)**, (**c**) Main overexpressed (**b**) or downexpressed (**c**) proteins in purified cytoplasmic fractions of hCPC compared with hMSC, analyzed by LF proteomics (*n* = 3); code: ++++ , indicates > 10 peptides; +++ , 5–10 peptides; ++, 2–4 peptides; + , 1 peptide and + / − , 0–1 peptides. (**d**) PANTHER GO-Slim Biological Processes analysis of overexpressed cytoplasmic proteins in hCPC. (**e**) Comparative RT-qPCR expression analysis of *P4HA1* in the three independent isolates of hCPC (hCPC 1–3), two human fibroblasts (HDF1 and F3) and two hMSC isolates (MSC19 and MSC45). Assays were performed three times and data are expressed as mean ± SD; black lines summarize p-values (*** < 0.002) for hCPC vs. fibroblasts or hMSC (one-way ANOVA analysis of variance followed by the Bonferroni correction for multiple comparison). (**f**) Western blot analysis of P4HA1 in the three independent isolates of hCPC (hCPC 1–3), human dermal fibroblasts (HDF1) and an hMSC isolate (MSC19); tubulin was used as a loading control. 'Full-length blots/gels' are presented in Supplementary Figure ﻿S5 online.

Whole label-free (LF) proteomic analyses of hCPC and hMSC^34^ yielded 1,260 and 1,176 cytoplasmic proteins, respectively; 95% of which could be mapped onto a GO category. Ingenuity Pathway Analysis (IPA) of the cytoplasmic hCPC subproteome is shown (Fig. 1a). Cytoplasmic fractions of hCPC and hMSC were obtained and analyzed first by LF proteomics; 748 and 707 cytoplasmic proteins were identified in hCPC and hMSC, respectively (Supplementary Fig. S1 online). Among the cytoplasmic proteins expressed more differentially in hCPC, we identified 11 upregulated proteins, including 3'-phosphoadenosine 5'-phosphosulfate synthase 2 (PAPSS2), procollagen-lysine, 2-oxoglutarate 5-dioxygenase 1 (PLOD1) and prolyl 4-hydroxylase, alpha polypeptide I/II, (P4HA1 and P4HA2) (Fig. 1b). We also identified 5 moderately downregulated proteins in hCPC, including aspartate beta-hydroxylase (ASPH) and insulin-like growth factor mRNA binding protein 2 (IGFBP2) (Fig. 1c). PANTHER GO-Slim analysis of biological processes using the upregulated hCPC cytoplasmatic proteins clearly indicated an over-representation of muscle contraction-associated proteins in hCPC (Fig. 1d) and PANTHER Pathway analysis showed an over-representation of cytoskeletal regulation by Rho GTPases (Supplementary Fig. S1 online).

To validate the proteomic data, we compared *P4HA1* and *ASPH* expression in hCPC, hMSC and fibroblasts by RT-qPCR analysis. The data confirmed *P4HA1* overexpression in hCPC (Fig. 1e). This was also confirmed by western blotting, although the difference in hCPC P4HA1 expression was less pronounced when compared with human fibroblasts, and no differences were evident when compared with hMSC (Fig. 1f). When we analyzed the gene expression of *ASPH*, we found that it was not downregulated in hCPC (Supplementary Fig. S1 online), as inferred from the proteomic analysis (Fig. 1﻿c), suggesting a post-transcriptional regulation in hCPC. Additionally, we discarded the possibility that the expression differences found would be associated with the cardiac origin of the hCPC analyzed. We compared their differential expression with total human heart samples confirming all results with the the sole exception of *CDH5* (Supplementary Fig. S1 online).

### Comparative analysis of the human CPC nuclear compartment

**Figure 2:**
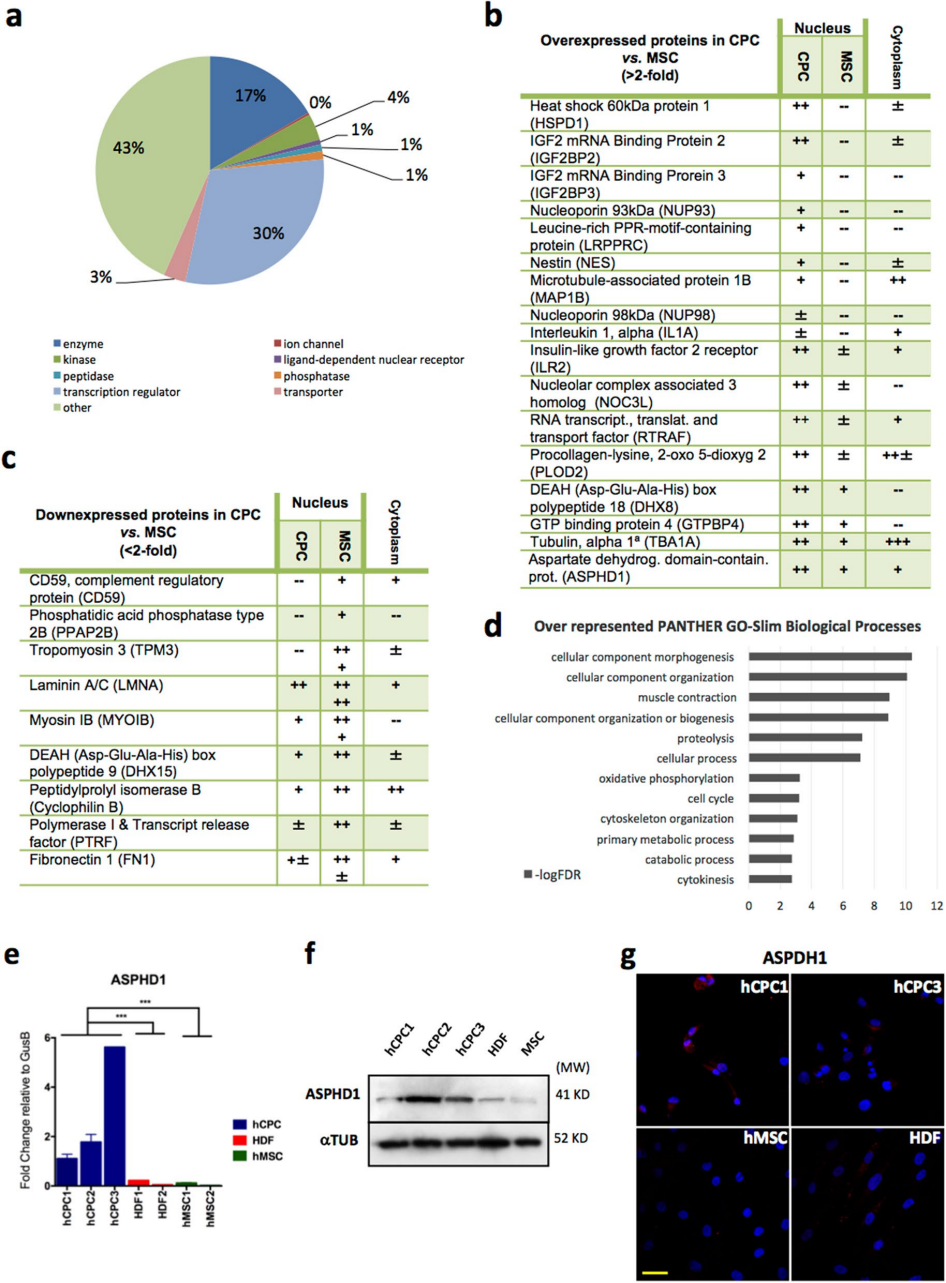
Comparative analysis of hCPC nuclear compartment. (**a**) Ingenuity pathway analysis (IPA) of the nuclear hCPC subproteome obtained by whole label-free (LF) proteomics; (**b**), **(c**). Main overexpressed (**b**) or downregulated (**c**) proteins in purified nuclear fractions of hCPC compared with hMSC, analyzed by LF proteomics (*n* = 3); code: +  +  + , indicates 5–10 peptides; +  + , 2–4 peptides; + , 1 peptide, + / − , 0–1 peptide and –-, no peptide detected. In parallel, comparative levels of the indicated proteins in cytoplasmic extracts are also indicated. (**d**) PANTHER GO-Slim Biological Processes analysis of overexpressed nuclear proteins in hCPC. (**e**) Comparative RT-qPCR expression analysis of *ASPHD1* in the three independent isolates of hCPC (hCPC 1–3), two human fibroblasts (HDF1 and HDF2) and two hMSC isolates (MSC19 and MSC45). Assays were performed three times and data are expressed as mean ± SD; black lines summarize p-values (*** < 0.002) for hCPC *vs.* fibroblasts or hMSC (One-way ANOVA analysis of variance followed by the Bonferroni correction for multiple comparison). (**f**) Western blot analysis of ASPHD1 in the three independent isolates of hCPC (hCPC 1–3), human dermal fibroblasts (HDF1) and hMSC (MSC19) isolate; tubulin was used as a loading control.'Full-length blots/gels' are presented in Supplementary Figure S4 online. (**g**) Comparative immunofluorescence analysis of ASPHD1 (red) in hCPC (hCPC 1 and 3), hMSC (MSC19) and human dermal fibroblasts (HDF); nuclei were counterstained with DAPI (blue). Bar, 20 μm.

Analysis of the whole LF proteome in hCPC and hMSC yielded 446 and 514 nuclear proteins, respectively^34^; 95% of which could be included in a GO category. IPA analysis of the nuclear hCPC subproteome is shown (Fig. 2a). LF-proteomic analysis of purified nuclear fractions of hCPC and hMSC rendered 369 and 348 proteins, respectively (Supplementary Fig. S1 online). To confirm the proteins identified in the nuclear fraction, we compared with their representation in the cytoplasm (Fig. 2b,c). The comparative proteomic analysis of hCPC *versus* hMSC nuclear purified fractions revealed the potential differential expression of 27 proteins in hCPC (Fig. 2b,c). Of the more clearly overexpressed proteins in the hCPC nuclear compartment only IMP3 (also known as IGF2BP3), nestin and IL1A (Fig. 2b) also showed a parallel significant increase in mRNA expression in hCPC relative to hMSC. In addition, hCPC expressed lower nuclear levels of several proteins including Polymerase I and Transcript release factor (PTRF) (Fig. 2c); previous RNAseq studies^34^ confirmed all dentified proteins. PANTHER GO-Slim analysis of biological processes using the upregulated hCPC nuclear proteins indicated a strong involvement in cellular component morphogenesis and organization, as well as muscle contraction (Fig. 2d). PANTHER Pathway analysis also revealed an important representation of the ubiquitin proteasome pathway (Supplementary Fig. S2 online).

Aiming to validate the proteomics nuclear data we evaluated the expression of ASPHD1 and PTRF, which were up- and down-regulated, respectively, in hCPC *versus* hMSC, by proteomics. RT-qPCR analysis confirmed a significant differential expression of *ASPHD1* in all hCPC isolates (hCPC1–3) in comparison with hMSC and fibroblasts (Fig. 2e). The preferential expression in hCPC was also confirmed by western blotting and by immunofluorescence (Fig. 2f,g). By contrast, *PTRF* downregulation in hCPC was not confirmed by RT-qPCR (Supplementary Fig. S2 online), suggesting again a relevant post-transcriptional regulation.

Because a very significant fraction of regulatory nuclear proteins is expressed at low levels, below the detection limits of proteomics, we validated by RT-qPCR several transcriptional factors found up- and down-regulated, by RNAseq in hCPC compared with hMSC^34^. *GATA4, SOX17, WT1* and *GATA2* were robustly overexpressed in hCPC in comparison with hMSC (Supplementary Fig. S2 online). The expression levels of *TBX3* and *MEF2C* were also significantly higher in hCPC than in hMSC, but less pronounced. We also confirmed that *HOXD8* and *HOXA10* were barely expressed by hCPC in comparison with hMSC (Supplementary Fig. S2 online).

### IL1A is a dual-function cytokine in hCPC

Two of the most differentially expressed genes in hCPC were *IL1A* (as mentioned above) and *IL1B*, whose overexpressions were also validated by RNAseq^34^. IL1A was found over-represented by comparative LF-proteomic analysis in the nuclear compartment of hCPC compared with hMSC (Fig. 2b). IL1A is produced as a precursor protein that yields a mature form and an N-terminal propeptide, containing a nuclear localization sequence that allows access to the nuclear compartment. In this way, IL1A is a well established “dual-function cytokine” that plays a role in the nucleus, independently of its classical extracellular mediated effects^39–41^.

**Figure 3:**
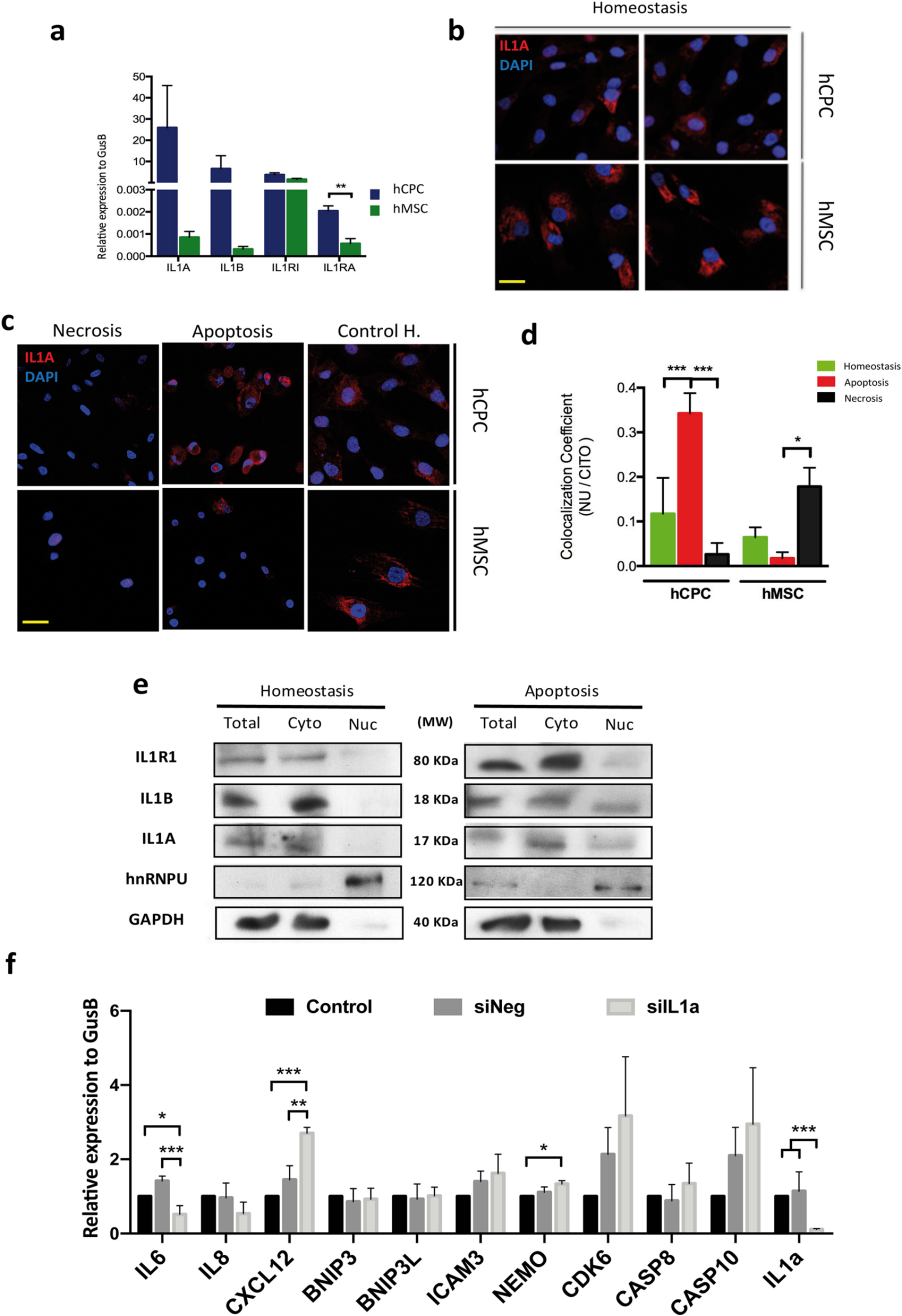
Comparative functional evaluation of IL1A in hCPC. (**a**) Comparative RT-qPCR expression analysis of *IL1A, IL1B, IL1R1* and *IL1RA* in hCPC1 compared with hMSC (MSC19). (**b**) Immunofluorescence analysis of IL1A expression (red) in hCPC1 compared with hMSC (MSC19), in homeostasis; nuclei were counterstained with DAPI (blue). Bar, 20 μm. (**c**) Comparative immunofluorescence analysis of IL1A expression (red) in hCPC1 and MSC19, in homeostasis or after induction apoptosis or necrosis; nuclei were counterstained with DAPI (blue). Bar, 20 μm. (**d**) Quantification by immunofluorescence of nuclear/cytoplasmic location of IL1A in hCPC1 and MSC19, comparing homeostasis and after apoptosis or necrosis induction; co-localization of IL1A with DAPI signal was compared with cytoplasmic pool; fluorescence intensity was measured using ImageJ software (NIH, Bethesda, MD). (**e**) Representative western blot analysis of IL1R1, IL1A and IL1B expression in purified cytoplasmic (Cyto) and nuclear (Nuc) fractions of hCPC1, in homeostasis (left) or after induction of apoptosis (right). GAPDH and hnRNPU were used as internal controls of cytoplasmic and nuclear fractions, respectively; lower contrast in these blots is caused by the higher intensity signal of these proteins. 'Full-length blots/gels' are presented in Supplementary Figure S5 online. (**f**) Target evaluation of IL1A in hCPC subjected to oxidative-mediated apoptosis. A panel of candidate genes, previously reported to be involved in apoptosis or immunoregulation, were evaluated by RT-qPCR in hCPC where IL1A was downregulated (siIL1A) in comparison with negative control hCPC (siNeg) and untransfected hCPC cells (Control). *IL1A* was confirmed to be significantly downregulated (> 90%). Assays were performed three times and data are expressed as mean ± SD; black lines summarize p-values (*** < 0.002; ** < 0.02; * < 0.05; one-way ANOVA analysis of variance followed by the Bonferroni correction for multiple comparison).

To validate the proteomic analysis, we assessed the expression of *IL1A* and *IL1B* by RT-qPCR in hCPC and hMSC. The results clearly confirmed the overexpression of *IL1A* and *IL1B* in hCPC (6,788- and 1,409-fold, respectively, Fig. 3a). We also tested the expression of other members of the IL1 signaling pathway. A significant increase (3.22-fold change) was found for the expression of the natural antagonist of IL1R1 (*IL1RA*) (Fig. 3a). Other main members of the IL1 pathway, IL1 receptor *(IL1RI)*, *IL38* and the secondary IL1 receptor (*IL1R2*) were, or not differentially expressed (*IL1RI)* or not detected. We next evaluated whether IL1A could acts as a dual-function cytokine in hCPC, as has previously been described in other cell lineages^42^. We analyzed in hCPC the behavior of *IL1A*, *IL1B* and *IL1RI* expression in response to apoptosis- or necrosis-promoted by oxidative stress (see Methods). We found that *IL1RI* expression was equivalently and moderately reduced (~ 25%) by both treatments (preferential apoptosis or necrosis). *IL1A and IL1B* expression were also decreased by both treatments, but to a much greater extent; *IL1A* expression was more pronouncedly reduced (90–95% reduction) when necrosis was induced (Supplementary Fig. S3 online).

Immunofluorescence analysis of IL1A, with an antibody against the full size protein, revealed a highly preferential cytoplasmic location in basal conditions (homeostasis; Fig. 3b). However, while hCPC showed significantly higher levels of *IL1A* mRNA expression than hMSC (Fig. 3a), the latter showed higher levels of IL1A protein (Fig. 3b), suggesting an important lineage-specific post-transcriptional regulation. In homeostasis the results confirmed an almost exclusive cytoplasmic location for all proteins analyzed. The fraction of nuclear IL1A detected by proteomics (Fig. 2b) thus seems to be minor. Of note, IL1A behaved differently to the two stmuli in hCPC and hMSC, as evaluated by immunofluorescence. Upon induction of apoptosis, IL1A protein was significantly upregulated in hCPC, whereas it was clearly reduced in hMSC (Fig. 3c); induction of necrosis provoked a major loss of IL1A in both cell types (Fig. 3c). Quantification of nuclear *versus* cytoplasmic localization by immunofluorescence of IL1A in hCPC, comparing homeostasis with the induction of apoptosis or necrosis, revealed a significant increase in the nuclear location of IL1A (co-localization with DAPI signal) after the induction of apoptosis (Fig. 3d). The opposite was found in hMSC where co-localization of IL1A with the nuclear compartment was poorer upon apoptosis induction, although it was augmented after necrosis induction (Fig. 3d). All these results suggest a high variable cell-type linage behavior.

We therefore performed western blotting of subcellular compartments in hCPC subjected to apoptosis compared with homeostasis (Fig. 3e); in necrotic cells, expression of the three proteins was quite low and difficult to quantify because of the strong loss of cellular content. In agreement with the immunofluorescence study (Fig. 3c), a substantial fraction of IL1A and IL1B was found in the nuclear compartment (Fig. 3e) of apoptotic cells whereas the subcellular localization of IL1RI was barely unchanged by the induction of apoptosis. Densitometric analysis of the representative western experiment shown (Fig. 3e) yielded an important nuclear/cytoplasmic ratio increment for IL1A and IL1B, in apoptotic cells compared with cells in homostasis, and a modest variation on IL1RI (Supplementary Fig. S3 online).

Furthermore we analyzed whether IL1A could be regulating transcription during early apoptosis. For that we have compared expression level of several genes involved in apoptosis or inflammation in hCPC cells knockdown (~ 20%) for IL1A (hCPC-siIL1A) in comparison with control hCPC cells (hCPC-siNeg) or negative-control tranfected cells (Supplementary Fig. S3 online); all cell populations were exposed to H_2_O_2_ during a short period (500 μM; 5 h) (Fig. 3f). Results indicated that although several genes involved in apoptosis (BNIP3, BNIP3L and ICAM3), are non-affected by IL1A knockdown, CDK6, Casp8, Casp10 showed a clear trend for overexpression in IL1A knockdown hCPC-siIL1A although the differences did not result statitistically significant. CXCL12 and NEMO were clearly overexpressed in for hCPC-siIL1A compared with control hCPC-siNeg. Finally, expression of IL6 was also clearly downregulated in hCPC-siIL1A. Altogether, these data reinforced that idea that IL1A could be acting as dual-cytokine in hCPC with a potential role in the transcriptional regulation in apoptosis.

Given the known immunoregulatory capacity of hCPC^32–34^ and their definition as an MSC-like cell subpopulation^33^, it is possible that IL1A could play a role in hCPC in homeostasis. We thus evaluated whether IL1A could contribute to the immunoregulatory capacity of hCPC. Thus, we co-cultivated phytohemagglutinin-stimulated human CD3 T cells with control hCPC, hCPC siIL1A, or hCPC siNeg. All cell populations (hCPC, hCPC siIL1A and hCPC siNeg) demonstrated similar immunoregulatory capacity at the higher cell doses analyzed (1:10–1:20), which was lost when lower doses were evaluated (1:40). Therefore no significant changes in immunoregulatory capacity were found (Supplementary Fig. S3 online), indicating that IL1A seems not to have a relevant role in the T cell immunoregulatory capacity of hCPC.

### Functional evaluation of IMP3 in hCPC in homeostasis and in response to oxidative damage

**Figure 4:**
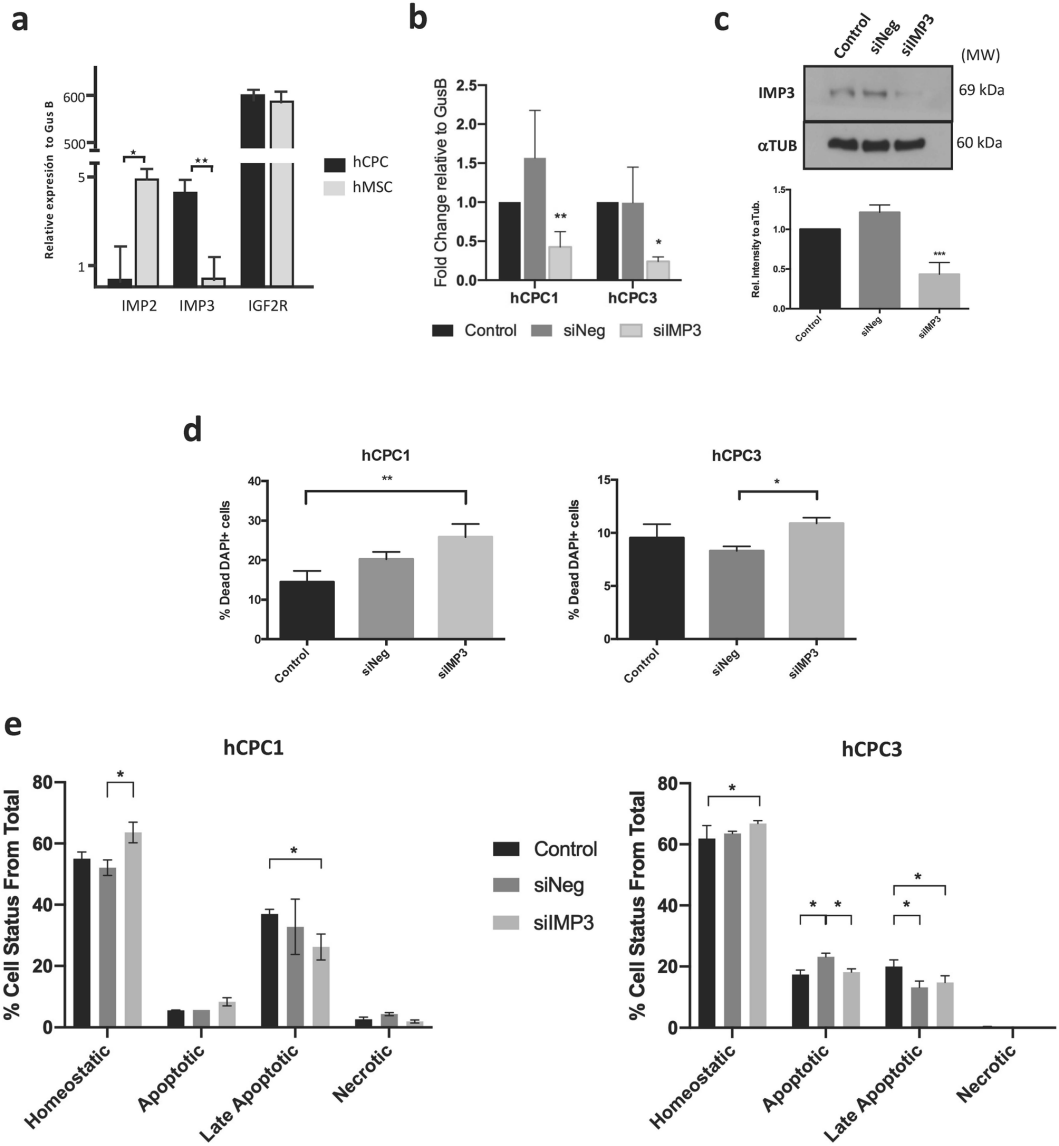
Functional evaluation of IMP3 in hCPC in homeostasis and in response to oxidative damage. (**a**) Comparative RT-qPCR expression analysis of *IGF2R, IMP2* and *IMP3* in hCPC1 and MSC19. (**b)**, (**c**) Confirmation of downregulation of IMP3 in hCPC (1,3) transfected with sIMP3 compared with a negative control (siNeg) and untransfected control cells (control), by RT-qPCR relative to the expression of *GusB* (**b**) and western blot (**c**); bottom panel corresponds to the quantification, relative to tubulin, on the representative western blot (upper panel); 'full-length blots/gels' are presented in Supplementary Figure S5 on line. All samples were analyzed 48 h post-transfection. (**d**) Evaluation of cell viability in hCPC (1,3) transfected with sIMP3 compared with a negative control (siNeg) and untransfected control cells (control), by DAPI staining, evaluated 48 h post-transfection. (**e**) Analysis of the effects of IMP3 downregulation on hCPC (1,3) response to oxidative damage induced by H_2_O_2_. hCPC control, siIMP3- or siNeg-transfected cells were exposed to H_2_O_2_ (500 μM) for 48 h; then cultures were stained with AnnexinV/propidium iodide (Anex.V/P.I.) and homeostatic viable (Anex.V − /PI −), apoptotic (Anex.V + /PI −), late apoptotic (Anex.V + / PI +) or necrotic (Anex.V −/PI +) cells were quantified by cytometry. Assays were performed three times and data expressed as mean ± SD; black lines summarize p-values (*** < 0.002, ** < 0.02, * < 0.05; one-way ANOVA analysis of variance followed by the Bonferroni correction for multiple comparison).

IGF2 is the predominant form of IGF in humans^43^ and it binds to insulin-like growth factor 1 receptor (IGF1R), insulin-like growth factor 2 receptor (IGF2R; CD222) and the insulin receptor A isoform (IR-A). It seemed interesting that in addition to IGF2R, two additional members of the IGF2 pathway (insulin-like growth factor mRNA binding proteins 2 and 3; IMP2/IMP3) were identified as over-represented in the hCPC nuclear subproteome by LF-proteomics (Fig. 2b). RT-qPCR analysis validated the high levels of *IMP3* expression in hCPC *versus* hMSC (> 40-fold overexpression), but the opposite was observed for *IMP2* (Fig. 4a).

IMP3 belongs to a family of mRNA-binding proteins that bind to multiple mRNAs in mammalian cells, including IGF2^44^. Based on previous literature^43^, we hypothesized that high levels of IMP3 would lead to a decrease in the autocrine bioavailability of IGF2, reducing the potential signaling through IGF2R and triggering senescence/apoptosis. We first analyzed the impact of *IMP3* knockdown in two independent hCPC isolates. Cells transfected with siIMP3 showed significantly reduced levels of IMP3 when compared with negative control or non-transfected control cells, analyzed both by RT-qPCR and western blotting (Fig. 4b,c). *IMP3* silencing did not affect negativelly hCPC viability, 48 h post-transfection; in fact, IMP3-silenced cells showed a moderate increase in viability (Fig. 4d). We then analyzed the effects of *IMP3* silencing on the response of hCPC to oxidative stress (500 μM H_2_O_2_, during 48 h) and evaluating apoptotic and necrotic cells with the Annexin V/propidium iodide (Supplementary Fig. S4 online). Neither of the two hCPC populations tested showed any remarkable difference in the percentages of homeostatic, apoptotic, late apoptotic or necrotic cells (Fig. 4e). Thus, IMP3 does not seem to play a critical role in the regulation of hCPC response to oxidative stress-mediated apoptosis.

**Figure 5:**
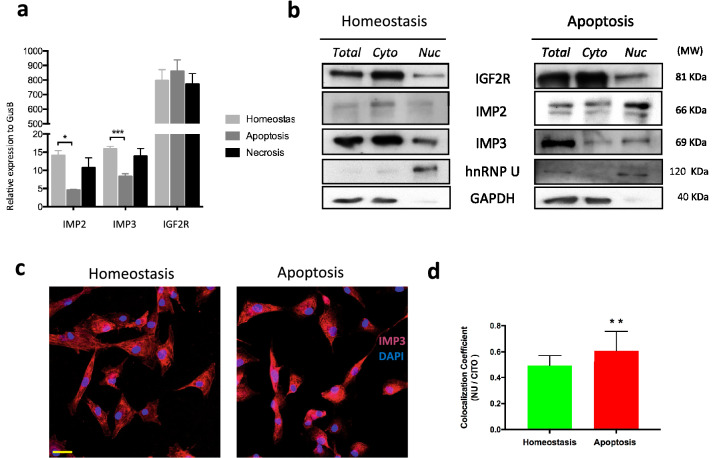
Evaluation of IMP3 expression and subcellular localization after apoptosis in hCPC. (**a**) Comparative RT-qPCR expression analysis of *IMP2*, *IMP3 and IGF2R* in hCPC1 in homeostasis and after the induction apoptosis and necrosis. Assays were performed three times and data are expressed as mean ± SD; black lines summarize p-values (** < 0.02; * < 0.05; one-way ANOVA analysis of variance followed by the Bonferroni correction for multiple comparison). (**b**) Representative western blot analyses of IGF2R, IMP2 and IMP3 expression in purified cytoplasmic (Cyto) and nuclear (Nuc) fractions of hCPC1 in homeostasis **(**left panel) or subjected to apoptosis (right panel). GAPDH and hnRNPU were used as internal controls of cytoplasmic and nuclear fractions, respectively; lower contrast in these blots is caused by the higher intensity signal of these proteins. 'Full-length blots/gels' are presented in Supplementary Figure S5 online. (**c**) Comparative immunofluorescence analysis of IMP3 expression (red) in hCPC1, in homeostasis or after induction apoptosis; nuclei were counterstained with DAPI (blue); Bar, 20 μm. (**d**) Quantification by immunofluorescence of nuclear/cytoplasmic location of IMP3 in hCPC1, comparing homeostasis and after apoptosis induction**;** co-localization coefficient of IMP3 with DAPI signal was compared with cytoplasmic pool; fluorescence intensity was measured using ImageJ software (NIH, Bethesda, MD). Assays were performed three times and data are expressed as mean ± SD; black lines summarize p-values (** < 0.02; t-test analysis for data with paired standard desviation).

Although IMP3 seems not to be essential for apoptotic responses we investigated IMP3 regulation in hCPC damage responses. We first studied the impact of apoptosis or necrosis induction on the transcriptional activity of *IMP3, IMP2* and *IGF2R*, and their subcellular localization. Neither apopotosis nor necrosis affected *IGF2R* expression; however, the induction of apoptosis (but not necrosis) promoted a significant decrease of *IMP3* and *IMP2* transcription in hCPC (Fig. 5a). Western blotting of hCPC showed that IGF2R and IMP3 were expressed at similar levels whereas IMP2 was expressed at apparently lower levels (Fig. 5b). Analysis of nuclear and cytoplasmic fractions by western blotting confirmed that a substantial fraction of IMP3, and to a lesser extent IMP2, was found in the nuclear fraction in homeostasis (Fig. 5b, left panel). After induction of apoptosis, both IMP2 and IMP3 showed an increased presence in the nuclear compartment with respect to the cytoplasmic compartment, whereas IGF2R was unchanged (Fig. 5b; right panel); densitometric analysis of the representative western blot shown (Fig. 5b) yield an increase in nuclear IMP2 and IMP3 of 8.5-fold and 13-fold, respectively, in apoptotic cells compared with cells in homeostasis (Supplementary Fig. S4 online). These results were confirmed by immunofluorescence (Fig. 5c,d). We analyzed the nuclear *versus* cytoplasmic localization of the IMP3 fluorescent signal and we confirmed that, upon induction of apoptosis, the nuclear pool of IMP3 singnificantly increases (co-localization coefficient referred to DAPI signal) (Fig. 5d).

Thus, apoptosis induction in hCPC triggers a significant decrease in *IMP2* and *IMP3* transcription, concomitant with an enrichement of both proteins in the nuclear compartment. These results suggest a non-essential role of IMP3 in gene expression regulation upon induction of oxidative stress-mediated apoptosis.

### IMP3 regulates proliferation and migration of hCPC

**Figure 6:**
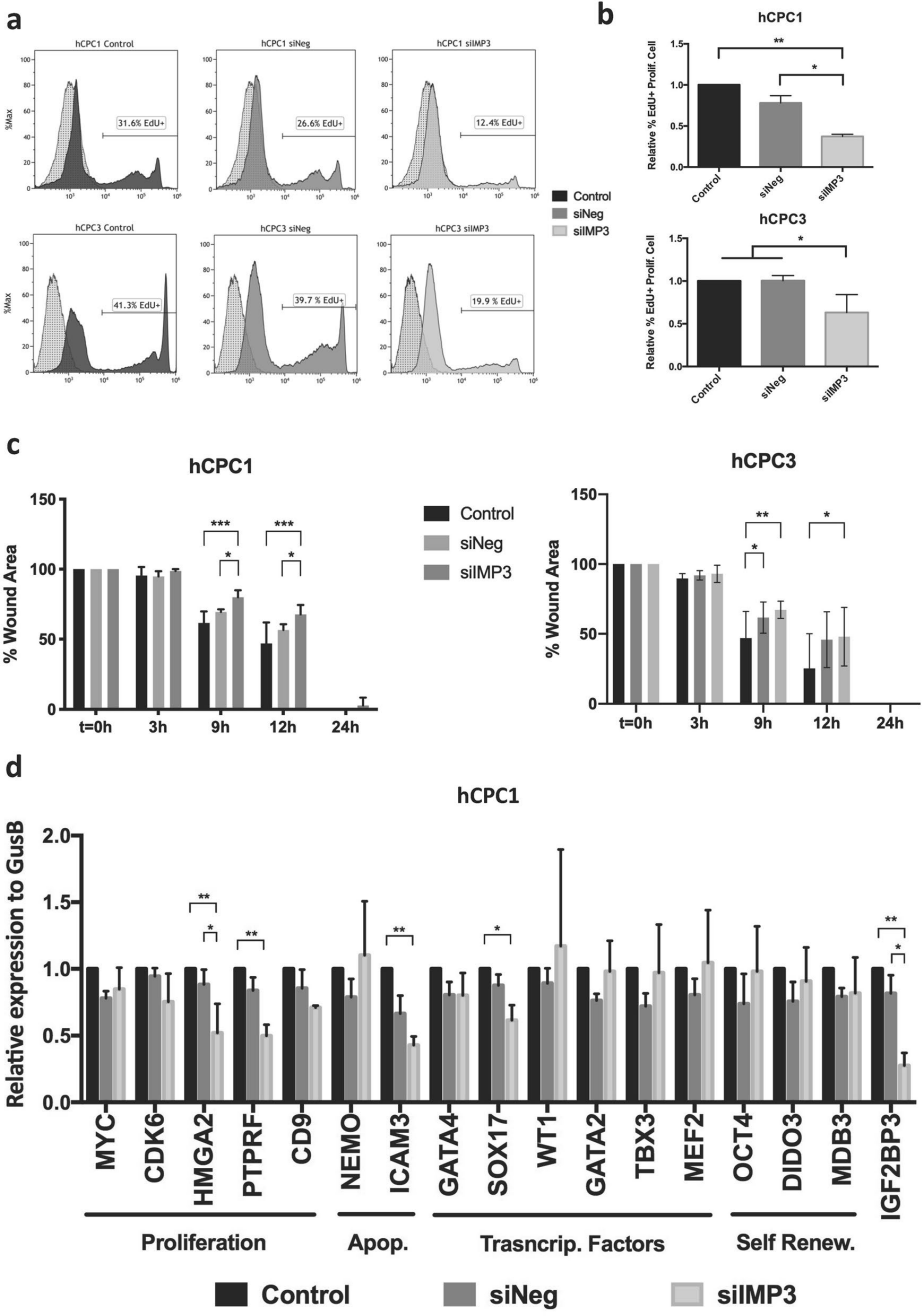
IMP3 regulates proliferation and migration of hCPC. (**a**), (**b**) Analysis of the effect of *IMP3* downregulation on hCPC proliferation rate, estimated by EdU (5-ethynyl-2’-deoxyuridine) incorporation during 12 h. hCPC (1,3) transfected with sIMP3 were compared with a negative control (siNeg) and untransfected control cells (Control), were evaluated 48 h post-transfection by flow cytometry. (**a**) Representative histograms. (**b**) Percentage of EdU + cells. (**c**) Evaluation of the effect of IMP3 downregulation (siIMP3) on the wound-healing capacity of two independent hCPC isolates (1,3) compared with a siNeg and control non-transfected cells 48 h post-transfection. Evolution of the wounded area was monitored during 24 h. (**d**) Target evaluation of IMP3 in hCPC. A panel of candidate genes, previously reported to be regulated by IMP3 in heterologous models or described as preferentially expressed in hCPC, were evaluated by RT-qPCR; genes are related to proliferation (*MYC, CDK6, HMGA2, PTPRF,* and *CD9*), apoptosis (*NEMO* and *ICAM3*), transcriptional factors (*GATA4, SOX17, WT1* and *GATA2*) and self-renewal (*OCT4, DIDO3* and *MBD3*). *IMP3* was confirmed to be significantly downregulated (> 70%). IMP3 knockdown hCPC cells (siIMP3) were compared with a siNeg and non-transfected control cells. Assays were performed three times and data expressed as mean ± SD; black lines summarize p-values (*** < 0.002, ** < 0.02, * < 0.05; one-way ANOVA analysis of variance followed by the Bonferroni correction for multiple comparison).

Analysis of proliferation in *IMP3*-silenced cells and controls estimated by EdU (5-ethynyl-2’-deoxyuridine) incorporation during 12 h and 48 h post-transfection revealed that the knockdown of *IMP3* significantly reduced proliferation in hCPC1 cells (about twofold) and a similar effect was found in hCPC3 cells (Fig. 6a,b). Thus, IMP3 seems not to be relevant for survival, but is likely involved in hCPC proliferation regulation.

We then evaluated the potential implication of IMP3 in cell motility, as previously proposed^45^ using wound-healing assays. Monolayers of hCPC cells silenced or not for *IMP3* were compared in their capacity to repair a wound during 24 h, as described^34^. As shown in Fig. 6c, both *IMP3*-silenced hCPC isolates demonstrated a statistically significant delay (at 9–12 h) in wound healing, albeit with different kinetics (Fig. 6c).

Finally, we analyzed a panel of candidate target genes previously reported to be regulated by IMP3 in heterologous models, or described as preferentially expressed in hCPC^34,36^. We used RT-qPCR to compare the levels of gene expression in hCPC silenced for *IMP3* knockdown (hCPC-siIMP3) in comparison with control hCPC cells or negative-control tranfected cells (hCPC-siNeg). Figure 6d shows the results obtained with isolate hCPC1, which showed more robust *IMP3* silencing.

Concerning genes involved in proliferation, we found that *PTRF* (also known as Cavin1 or Cavin-1) and *HMGA2* (high-mobility group AT-hook 2) were significantly downregulated in hCPC- siIMP3 cells (~ 50%). *c-MYC, CDK6* and *CD9* were moderately but not significantly downregulated (< 20%). In relation to genes involved in apoptosis, we found that *ICAM3* expression was significantly reduced (~ 60%) in *IMP3*-silenced cells, but unexpectedly not *NEMO* (inhibitor of nuclear factor kappa B kinase subunit gamma).

We also tested the consequence of *IMP3* silencing for the expression of a small panel of transcriptional factors previously (Supplementary Fig. S2 online) defined in hCPC. All them, except *SOX17* expression (~ 40% reduction), did not modify the expression in hCPC-siIMP3. These results suggest that IMP3 might have a modest role in regulating hCPC fate-genes by regulation of *SOX17*. We additionally analyzed the potential impact of *IMP3* knockdown on several genes associated in other cell types with self-renewal, such as *OCT4*, *DIDO3* and *MBD3*^35,37,46^. The expression of all three genes was unaffected by *IMP3* silencing (Fig. 6d). These results suggest that IMP3 seems not to be mainly involved in the regulation of the undifferentiated state of hCPC. A similar analysis using hCPC3 yielded essentially identical but non-significant results.

Finally, because nuclear IL1A also regulates cell proliferation and migration^39,40^, we sought to evaluate the potential involvement of IMP3 in the regulation of the IL1 pathway. *IMP3*-silenced hCPC and controls were evaluated by RT-qPCR 48 h post-transfection. Compared with control hCPC, *IMP3*-silencing failed to affect *IL1A* expression but enhanced *IL1B* expression (Supplementary Fig. S4 online). *IL1RA* and *IL1R1* were also unaffected by *IMP3*-silencing (Supplementary Fig. S4 online), indicating that IMP3 is likely not involved in the regulation of IL1 pathway in hCPC.

## Discussion

Cardiosphere-derived cells (CDC) and c-kit^pos^ hCPC have been previously characterized and evaluated in preclinical studies, demonstrating modest therapeutic efficacy in acute ischemia models^22–25^. Based on previous studies, hCPC were defined as an hMSC-like population with confirmed immunoregulatory capacity^33,34,36^. Considering the promising, but not statistically significant, results of the clinical studies based on these cells^37,38^ a more detailed description of hCPC populations might lead to a better understanding of their mechanism of action and, ultimately, the development of more effective treatments.

Analysis of the most relevant cytoplasmic proteins over-represented in hCPC suggests that these cells might be well suited to mount an effective response to hypoxia, demonstrating also an active collagen metabolism. P4HA1 and P4HA2 are both overexpressed (P4HA1 > P4HA2) in the cytoplasmic compartment of hCPC, being both activated by hypoxia^47^. We also found PLOD1 and PLOD2 to be significantly overexpressed. P4HA1 and P4HA2 are required for collagen deposition, whereas PLOD2 is required for extracellular matrix stiffening and collagen fiber alignment. Furthermore, DHX9, an ATP-dependent helicase of double-stranded RNA and DNA-RNA complexes^48^, is highly upregulated during activation of quiescent cells to collagen-producing cells^49^. In the context of different adult stem cell compartments, CKAP4 and DHX9, both overexpressed in the hCPC cytoplasm, have been related to differentiation regulation^50^. CKAP4 is a nucleoplasmic shuttle protein that acts as a high-affinity receptor for antiproliferative factor (APF)^51^. DHX9 has been also proposed as a RISC-loading factor^52^. Concerning the proteins that were found moderately downregulated in hCPC cytoplasm, ASPH and TXNDC5 are also implicated in proliferation and cell motility regulation^53,54^. Altogether, hCPC might demonstrate an effective in response to hypoxia associated-damage showing an active remodeling of the extracellular matrix.

Regarding the proteins preferentially expressed in the hCPC nuclear compartment, we confirmed high levels of expression of several cardiogenic transcriptional factors such as *GATA4, SOX17, WT1, GATA2* and *TBX3* (Supplementary Fig. S2 online), with *GATA4* and *SOX17* more differentially expressed in comparison with hMSC. In addition, comparative proteomics analysis of enriched nuclear fraction yielded a panel of proteins more represented in hCPC than in hMSC. Among them, ASPDH1 that was also confirmed by RT-qPCR is poorly characterized. Finally, among the proteins under-represented in the hCPC nuclear compartment, it is noteworthy that levels of PTRF/cavin-1 have been directly associated with cell senescence. PTRF has been demonstrated to mediate in transcription pausing and termination, and the final dissociation of the transcription complex^55^.

Among the nuclear over-represented proteins in hCPC, IL1A and IMP3 were selected for further analysis. IL1A is a pro-inflammatory cytokine with multiple immune-regulatory functions. It is mainly expressed as a cell-associated form and not actively secreted in healthy tissue, but its membrane-associated form is critically involved in cell senescence^56^. IL1A is one of the four (IL1A, IL33, HMGB1 and S100) “dual-function cytokines” described in mesenchymal cells. These cytokines play a role in the nucleus independently of their extracellular-mediated effects, as a classical cytokines, and have been also called “damage-associated molecular pattern” molecules or alarmins^42^. Unlike IL1B, processed IL1A has a nuclear localization sequence and is trafficked to the nucleus, regulating cell proliferation and migration^40,41^. For example, in acute lymphocytic leukemia T cells, overexpression of the IL1A nuclear propeptide has been demonstrated to promote proliferation and reduce apoptosis, by NFkB and SP1 up-regulation^57^. Analyses of hCPC in homeostasis demonstrated a strong post-transcriptional regulation of *IL1A* mRNA and a highly preferential cytoplasmic location of IL1A. We found that IL1A is not related with the immunoregulation capacity of hCPC but, upon induction of apoptosis, IL1A was clearly upregulated and a substantial nuclear fraction was found; this behavior was not paralleled by hMSC. We also found a similar intracellular pattern for IL1B, although less pronounced. In this sense, IL1A knockdown in hCPC, follow by a short period of oxidative stress-associated apoptosis, demonstrated significant alterations in several apoptosis or inflammatory genes. Overall these results suggested that IL1A, and probably IL1B, could have dual-cytokine profile in hCPC, playing a role in the regulation of response to apoptosis.

IMP3 is an mRNA binding protein that, among other functions, regulates IGF2 expression^44^. In the context of cancer, there are numerous examples of the critical role of IMP3 favoring chemoresistance, aggressiveness and metastasis^58,59^. In neural and pancreatic cancer cells, IMP2- and IMP3-bound transcripts are localized in cytoplasmic RNA granules that accumulate in dendrites or membrane protrusions, where they are preferentially translated^60^. In pancreatic ductal adenocarcinoma cells, IMP3 modulates miRNA-mRNA interactions^61^. However, IMP3 binding could result both in an enhanced expression of the target mRNA^62^ or its destabilization^63^. Although different pathways and targets have been associated with the overexpression of IMP3 in cancer, few studies have addressed the role of IMP3 in healthy developmental processes; i.e. muscle growth is regulated by IMP3 levels, controlled by let-7b^64^ and adult megakaryocyte development is also under the control of IMP3, by regulating P-TEFb^45^.

hCPC in homeostasis show a clear overepresentation of IMP3, but not IMP2, in the nuclear compartment and induction of apoptosis provoked an enrichment in the nuclear compartment. *IMP3* knockdown reduced hCPC proliferation and migration capacity, although it had no obvious impact on viability. IMP3 has been found to promote cell migration in glioma by increasing the levels of p65 protein (RELA; subunit of NF-κB heterodimer), but without modifying transcript levels^65^. In glioblastomas IMP3 also promotes cell proliferation, migration and invasion by inducing epithelial-mesenchymal transition^58^.

Finally, we analyzed a panel of candidate target genes whose expression could be affected by the downregulation of IMP3 in hCPC. *cMYC, CD44 and CDK6* were demonstrated previously to be targeted by IMP3 in mixed lineage leukemia, enhancing the half-life of the transcripts^62^. Silencing of *IMP3* (hCPC-siIMP3 cells), however, resulted in a moderate and non-significant downregulation of these targets in hCPC. By contrast, silencing of *IMP3* led to a significant downregulation of *HMGA2* and *PTPRF.* HMGA2 is considered as an architectural transcription factor that is involved in growth regulation and tumorigenesis^46^. Interestingly, it has been demonstrated that IMP3 ribonucleoprotein complexes contain *HMGA2* mRNA, preventing miRNA-directed mRNA decay during tumor progression^66^. In addition, it has been recently demonstrated that HMGA2 controls both, proliferation and migration / metastasis, in colon cancer^67^; analyses of other HMGA2 candidate genes associated with cell migration (ARF6, ARHGEF4) rendered negative results. It is also worth noting that *HMGA2* mRNA is significantly overexpressed (17.8 fold) in hCPC *versus* hMSC^34^. By contrast, high PTRF expression levels correlate with an increased senescence in human fibroblasts^55^. Therefore, these data suggest that *PTRF* and *HMGA2* are regulated by IMP3 and, consequently, could be involved in hCPC proliferation/migration regulation.

In conclusion, we have compared, using a label-free proteomic approach, the differential cytoplasmic and nuclear compartments of human CPC (hCPC) versus human mesenchymal stem cells (hMSC) and fibroblasts. Globally, hCPC, with a clear cardiogenic transcriptional factor profile, are well suited to mount an effective response to hypoxia with active collagen metabolism. IL1A, characterized as a dual-function cytokine, seems to play a role in the regulation of the hCPC response to apoptosis caused by oxidative stress. Finally, IMP3 was demonstrated to be involved in hCPC proliferation and migration.

## Methods

### Ethical approval

Human CPC were obtained from human right atria appendage from adult donors, with no relevant cardiac pathology, and subjected to cardiac surgery with extracorporeal circulation; during the procedure, this tissue is normally discarded during cannulation. Human CPC were isolated from human myocardial samples by c-kit immunoselection, as described^32^. Procedures were approved by the hospital ethical committees (Hospital 12 de Octubre and Hospital Universitario Gregorio Marañón, Madrid, Spain) with the corresponding patient informed consents. All methods were carried out in accordance with relevant guidelines and regulations (R.D. 9/2014 and Orden SSI/2057/2014, which transpose the European Commission Directive 2012/39/UE). hCPC1-hCPC3 isolates have been previously characterized^34,36^.

### Cells and culture conditions

hCPC were maintained and expanded as previously indicated^34,36^, essentially under equivalent conditions to those used in the CAREMI clinical trial (EudraCT 2013-001358-81). See Supplementary "Methods" section online for details. All cells were expanded and manipulated (induction of oxidative damage and transfections) in an atmosphere of 3% O_2_/5% CO_2_, which mimics physiologic conditions and reduces the senescence evolution of the cultures^63^. Human bone marrow-derived MSC were obtained from the Inbiobank Stem Cell Bank (www.inbiobank.org) under specific regulations (R.D. 1301/2006). Human fibroblasts were purchased from the American Type Culture Collection (Manassas, VA; cat# CRL-2097), ScienceCell Research Laboratories (San Diego, CA; cat# 6300) and PromoCell (Heidelberg, Gemany; cat# C-12375 and C-12360). hMSC and fibroblasts were maintained and expanded under optimal conditions, previously described^34,36^, also in a 3% O_2_/ 5%CO_2_ atmosphere. A more detailed description can be found in expanded methods (Supplementary "Methods" section online details).

### Label-free proteomics analysis

hCPC3 protein levels were compared with those of hMSC19, essentially as previously described^34^. Cells were expanded to P7- P8, recovered and, after several washes in PBS, pellets (5–8 × 10^7^ cells) were collected. Subcellular cytoplasmic and nuclear protein fractions (see Supplementary "Methods" section online) were obtained using the Qproteome Cell Compartment Kit (Qiagen, Barcelona, Spain). Samples (~ 500 μg) were digested using an in-gel digestion protocol, as described^20^. Tryptic peptides were dissolved in 0.1% formic acid (FA) and loaded on a liquid chromatography-mass spectrometry (LC–MS/MS) system for online desalting on C18 cartridges and further analysis by LC–MS/MS, using a reverse-phase nanocolumn (75 μm inner diameter × 50 μm, 3 μm-particle size, Acclaim PepMap 100 C18; Thermo Fisher Scientific, San Jose, CA) in a continuous (0–30%) acetonitrile gradient consisting of B (90% acetonitrile, 0.5% formic acid), in 180 min, 30–43% in 5 min and 43–90% in 2 min. A ~ 200 nL/min flow rate was used to elute peptides from the nanocolumn to an emitter nanospray needle for real time ionization and peptide fragmentation onto an ion trap-orbitrap hybrid mass spectrometer (Orbitrap Elite, Thermo Fisher). Bioinformatic identification and analyses methods are described in Supplementary "Methods" section online. Relative representation of the different proteins identificated was estimated by peptide-counting; three replicas were analyzed for each comparison. When indicated pathway analysis with PANTHER software^68^ was carried out.

### RT-qPCR analyses

Total mRNA was isolated as described^33^. cDNA first strands were synthesized from total RNA (1 μg) with the SuperScript III First-Strand Synthesis System (Invitrogen). Genes of interest (see Supplementary "Methods" section online) were evaluated by quantitative RT-qPCR in a Mastercycler Ep-Realplex platform (Eppendorf, Hamburg, Germany), using Power SYBR Green reagents (Applied Biosystems, Foster City, CA). Cycle conditions were 95 °C for 10 min, followed by 40 cycles of 95 °C for 15 s and 60 °C for 1 min. Quantified gene expression values were normalized against those of *GUSB* or *GAPDH*. Supplementary Methods Table 1 section online includes the list of all primer sequences used.

### Western blotting

Cells were harvested in RIPA (radioimmunoprecipitation assay) lysis buffer, and equal amounts of lysate were separated by 10% SDS-PAGE. When indicated, cytoplasmic or nuclear fractions were obtained using the NE-PER Nuclear and Cytoplasmic Extraction kit (Thermo Fisher Scientific). Proteins were transferred to PVDF membranes using the iBlot Dry Blotting System (Invitrogen). After incubation with primary and secondary antibodies, signals were developed using an ECL kit (GE Healthcare, Uppsala, Sweden). Supplementary Methods Table 2 section online includes the list of all primary and secondary antibodies used.

### Immunofluorescence

Immunofluorescence protocols have been previously described in detail. Cells were fixed in 4% paraformaldehyde, permeabilized with 0.1% Triton-X100 (5 min, room temperature), blocked with blocking buffer (PBS with 5% BSA; 30 min, room temperature) and then incubated with primary antibodies in PBS/1% BSA (overnight, 4 °C). Slides were washed three times in PBS/1% bovine serum albumin (BSA) and incubated in PBS/1% BSA with appropriate secondary antibodies (1 h, room temperature). Washed cells (three times in PBS/1% BSA) were mounted in Prolong DAPI mounting medium (Invitrogen). Images were captured with a Zeiss LSM 700 or a Leica TCS SP5 confocal microscope. Supplementary Methods Table 2 section online includes the list of all primary and secondary antibodies used. Fluorescence intensity was measured using ImageJ software (NIH, Bethesda, MD) (https://imagej.net/software/imagej/).

### Gene silencing assays

hCPC were transfected in Opti-MEN medium (Gibco, Invitrogen) with 10 nM of small interfering RNA (siRNA) against IMP3 (siIMP3), IL1A (siIL1A) or an siRNA negative control (all provided by Origene Technologies, Rockville, MD) using Lipofectamine 2000 Reagent (Invitrogen, Thermo Fisher Scientific). Cells were maintained overnight with the transfection mix. RT-qPCR or western blotting within 24–48 h of transfection checked silencing efficiency. Functional effects were tested 48 h post-transfection, as maximum inhibition efficiency was confirmed at this time point.

### Viability, proliferation and apoptosis assays

To study cell viability, hCPC were detached with trypsin–EDTA 48 h post-transfection, labeled with DAPI (1/1000; Sigma-Aldrich) and quantified by flow cytometry on a FACS Canto 3L flow cytometer (BD Biosciences, San Jose, CA). For proliferation assays, 5-ethynyl-2’-deoxyuridine (EdU; 10 μM) was added to hCPC cultures 12 h prior to analysis. Proliferating cells were detected with the Click-iT Flow-Cytometry Kit (Thermo Fisher Scientific). For apoptosis analysis, cells were exposed to H_2_O_2_ (500 μM, during 5 h), then collected (including detached cells) and labeled at 4ºC for 15 min with AnnexinV-FITC (diluted 1:10) in the binding buffer provided by the manufacturer (ApoScreen® Annexin V Apoptosis Kit-FITC; Southern Biotech, Birmingham, AL). Labeled cells were washed with PBS/0.01% BSA and resuspended in 390 μL of binding buffer. Propidium iodide (50 μg/ml, Beckman Couler, Nyon, Switzerland) was added (1:40 dilution) for dual-staining and cells were analyzed by flow cytometry. DAPI and AnexinV/PI positive-cells were quantified on a FACS Canto 3L flow cytometer (BD Biosciences). When indicated, necrosis of hCPC was induced by a short heat treatment (10 min, 60ºC) of attached monolayers.

### Immunoregulation evaluation

Human peripheral mononuclear cells (MNC) were labeled with 1 µM carboxyfluorescein succinimidyl ester (CFSE CellTrace Cell Proliferation Kit; Molecular Probes, Invitrogen) and stimulated with 10 μg/mL phytohemagglutinin (Sigma-Aldrich) over three days, as described^69^. hCPC1 cells were used for the evaluation of hCPC immunoregulatory capacity on T cells. Cells were plated in triplicate at different cell densities (15 × 10^3^, 30 × 10^3^ or 60 × 10^3^) in 24-well plates and incubated at 37 °C for 16 h in an atmosphere of 3% O_2_/5%CO_2_. hCPC were transfected during 6 h with siIL1A or siNeg Control (10 nM) using 1 μL of Lipofectamine 2000 per well, in a final volume of 500 μL of Opti-MEN. The medium was then replenished with 100 μL of fresh DMEM-complete medium. CFSE-labeled MNC (6 × 10^5^) in 900 μL of RPMI-complete medium was added to the plates at different hCPC/MNC ratios (1:10, 1:20, 1:40) and incubated for 3 additional days. A comparative evaluation of viable (DAPI) CD3 + proliferative (CFSE +) cells was carried out by flow cytometry (Fortessa, BD Bioscience). Data were analyzed with ModFit LT (Verity Software House, Topsham, ME).

### Wound healing assay

For migration (scratch) assays, hCPC cells were cultured to confluence and starved in serum-free medium (24 h). The cell monolayer was then scraped with a pipette tip (t = 0 h) and cultures were monitored (t = 6–24 h) to evaluate their wound healing capacity. Images were acquired and migration rates were measured using ImageJ software (NIH, Bethesda, MD).

### Statistics

Assays were performed three times and data expressed as mean ± SD; black lines summarize p-values (*** < 0.002, ** < 0.02, * < 0.05) for hCPC *versus* fibroblasts or hMSC (one-way analysis of variance followed by the Bonferroni correction for multiple comparison).

## Supplementary Information

Supplementary Information.

## Data Availability

Mass spectrometry proteomics data are deposited in Peptide Atlas (http://www.peptideatlas.org/repository/) and are accessible through the PASS00827 accession number. All transcriptomic data related to this study are deposited in *Gene* Expression Omnibus (*GEO*) *database* repository (www.ncbi.nlm.nih.gov/geo/) and are accessible through the GSE84070 accession number.

## Abbreviations

B-CPC: Murine cardiac progenitor cells Bmi1^high^^+^
c-KIT, CD177: Receptor for stem cell factor
hCPC: Human cardiac progenitor cells
DAPI: 4',6-Diamidino-2-phenylindol
EdU: 5-Ethynyl-2’-deoxyuridine
hMSC: Human mesenchymal stem cells
HDF: Human dermal fibroblasts
H_2_O_2_: Hydrogen peroxide
IPA: Ingenuity Pathway Analysis
IL1A: Interleukin 1A, interleukin 1α
IL1B: Interleukin 1B, interleukin 1β
IMP2: IMP U3 Small Nucleolar Ribonucleoprotein 2, IGF2BP2
IMP3: IMP U3 Small Nucleolar Ribonucleoprotein 3, IGF2BP3
iTRAQ: Isobaric tags for relative and absolute quantitation
LF: Label-free
Log FC: Log_2_ fold-change
RNAseq: RNA sequencing
RT-qPCR: Quantitative polymerase chain reaction coupled to reverse transcription
siRNA: Small interfering RNA
