# Supplementary material for: Comparative proteomic analysis of nuclear and cytoplasmic compartments in human cardiac progenitor cells
Source: Sci Rep. 2022 Jan 7;12:146. doi: 10.1038/s41598-021-03956-8 (PMC8742012; doi:10.1038/s41598-021-03956-8)

Comparative proteomic analysis of nuclear and cytoplasmic compartments in human cardiac progenitor cells.

Guillermo Albericio, Susana Aguilar, José Luis Torán, Rosa Yañez, Juan Antonio López, Jesús Vázquez, Carmen Mora & Antonio Bernad *

Expanded METHODS

Cells and culture conditions

Human cardiac biopsies were obtained from patients suffering from an open-chest surgery, usually for valve replacement. Starting material was obtained from the right atria appendage, which is routinely removed in order to place the cannulae for the extracorporeal circulation. Tissue samples were minced into small pieces (<1 mm^3) and treated with collagenase type 2 (Worthington Biochemical Corporation, Lakewood, NJ, USA) for 3 cycles of 30 min each to obtain a cellular suspension and processed as previously described [1]. In brief, cardiomyocytes were removed by centrifugation and filtration using 40 µm cell strainers. Human cardiac progenitor cells (CPC) were purified from 3 human myocardial samples after immunodepletion of CD45-positive cells by c-kit immunoselection, following manufacturer recommendations. hCPC were maintained in DMEM/F12 and neurobasal medium (1:1), (both from Invitrogen; Madrid, Spain), supplemented with 10% fetal bovine serum embryonic stem cell-qualified (FBS ESCq, Invitrogen), 2 mM L-glutamine (Lonza; Belgium), penicillin-streptomycin (100 U/mL and 1000 U/mL, both from Lonza), 0.5X B27 supplement, 0.5X N2 supplement, 10 ng/mL bFGF and 0.5X ITS (all from Invitrogen), 30 ng/mL IGF-II and 20 ng/mL EGF (both from Peprotech; Neuilly-sur-Seine, France). Growth medium was exchanged weekly. hCPC were maintained in a 3% O_2/ 5%CO_2 atmosphere.

Human bone marrow-derived mesenchymal stem cells (MSC) were obtained from cadaveric bone marrow, harvested from brain-dead donors under the supervision of the Spanish National Transplant Organization (in Spanish, *Organización Nacional de Trasplantes*) under specific regulations (R.D. 1301/2006). Relatives gave written informed consent. Each sample donor was tested and found negative for HIV-1/2, hepatitis B-C, cytomegalovirus and mycoplasma. All cells were obtained from the Inbiobank Stem Cell Bank (www.inbiobank.org) following good manufacturing procedures based on ISO9001:2000.

hMSC and fibroblasts (obtained from Inbiobank) were maintained and expanded under optimal conditions, in low-glucose DMEM supplemented with 10% FBS (both from Sigma-Aldrich, Madrid, Spain), 2 mM L-glutamine (Lonza) and penicillin-streptomycin (100 and 1000 U/mL, respectively, Lonza), also in a 3% O_2/ 5%CO_2 atmosphere.

Proteomics analyses

Label-free proteomics analysis. hCPC3 and hMSC19 were used for proteomics analysis. Cells were expanded to passage 7–8, recovered (5–8 × 10^7) and washed several times in PBS. Subcellular cytoplasmic and nuclear protein fractions were obtained (n=3) using the Qproteome Cell Compartment Kit (Qiagen, Barcelona, Spain). When needed equivalent fractions were obtained from human fibroblasts (HDF).

Samples (~500 μg) were digested using an in-gel digestion protocol, as described [2]. Briefly, samples were resolved by conventional SDS-PAGE until the electrophoresis front entered 3 mm into the concentrating gel. The protein band containing the whole proteome was visualized by Coomassie staining, excised, cut into cubes, subjected to reduction conditions using 10 mM dithiothreitol (DTT), alkylated with iodoacetamide (50 mM), and digested (overnight 37ºC) with 60 ng/mL modified trypsin (Promega, Madison, WI) at a 12:1 protein:trypsin (w/w) ratio in 50 mM ammonium bicarbonate (pH 8.8) containing 10% acetonitrile. The resulting tryptic peptides were extracted by incubation in 12 mM ammonium bicarbonate pH 8.8 followed by 0.5% trifluoroacetic acid (TFA). TFA was added to a final concentration of 1% and peptides were desalted on C18 Oasis-HLB cartridges and dried. Tryptic peptides were dissolved in 0.1% formic acid (FA) and loaded on a liquid chromatography-mass spectrometry (LC-MS/MS) system for online desalting on C18 cartridges and further analysis by LC-MS/MS, using a reverse-phase nanocolumn (75 μm inner diameter × 50 μm, 3 μm-particle size, Acclaim PepMap 100 C18; Thermo Fisher Scientific, San Jose, CA) in a continuous (0–30%) acetonitrile gradient consisting of B (90% acetonitrile, 0.5% formic acid), in 180 min, 30–43% in 5 min and 43–90% in 2 min. A ~200 nL/min flow rate

was used to elute peptides from the nanocolumn to an emitter nanospray needle for real time ionization and peptide fragmentation onto an ion trap-orbitrap hybrid mass spectrometer (Orbitrap Elite, Thermo-Fisher). To increase proteome coverage, tryptic peptides were fractionated by cation exchange chromatography (Oasis HLB-MCX column; Waters Corp., Milford, MA), desalted and analyzed as above.

***Bioinformatics identification and analyses*.**

For peptide identification, MS/MS spectra were searched with the SEQUEST HT algorithm implemented in Proteome Discoverer 1.4.0.29 (Thermo Scientific). For database searching at the Uniprot database containing all sequences from human genome (March 6, 2013; 70024 entries; including common contaminants), search parameters were selected as follows: trypsin digestion with 2 maximum missed cleavage sites, precursor and fragment mass tolerances of 800 ppm and 1.2 Da, respectively, for the orbitrap Elite and 2 Da and 0.02 Da, respectively, for the QExactive, carbamidomethyl cysteine as fixed modification and methionine oxidation as dynamic modification. Peptide identification was validated using the probability ratio method [3] Martinez-Bartolomé et al., 2008) with an additional filtering for a precursor mass tolerance of 15 ppm [4]. False discovery rate (FDR) was calculated using inverted databases and a refined method for peptide identification using decoy databases [5].

References expanded Methods

1. Lauden, L., *et al.* Allogenicity of human cardiac stem/progenitor cells orchestrated by programmed death ligand 1. *Circ. Res*. **112**, 451-464 (2013).

2. Torán, J.L., et al. Definition of a cell surface signature for human cardiac progenitor cells after comprehensive comparative transcriptomic and proteomic characterization. Sci Rep. 9, 4647-4663 (2019).

3. Martínez-Bartolomé, S., et al. Properties of average score distributions of SEQUEST: the probability ratio method. *Mol. Cell Proteomics* **7**, 1135-1145 (2008).

4. Bonzon-Kulichenko, E., GarcÍa-Marques, F., Trevisan-Herraz, M., Vázquez, J. Revisiting peptide identification by high-accuracy mass spectrometry: problems associated with the use of narrow mass precursor windows. *J. Proteome Res*. **14**, 700-710 (2015).

5. Navarro, P., *et al*. General statistical framework for quantitative proteomics by stable isotope labeling. *J. Proteome Res.* **13**,1234-1247 (2014).

Table 1: List of RT-qPCR primers used in the study.

Gene	Sequence 5´-3´
IGF1	Fw: AGGAAGTACATTTGAAGAACGCAAGT<br>Rw: CCTGCGGTGGCATGTCA
IGF1R	Fw: AAAATGCTGACCTCTGTTACCTC<br>Rv: GCTTATTCCCCACAATGTAGTT
IGF2	Fw: CCGAAACAGGCTACTCTCCT<br>Rv: AGGGTGTTTAAAGCCAATCG
IGF2R	Fw: GAAGGTGAAGGTCGGAGT<br>Rv: GAAGATGGTGATGGGATTTC
IGF2BP2 (IMP2)	Fw: GTTCCCGCATCATCACTCTTAT<br>Rv: GAATCTCGCCAGCTGTTTGALO
IGF2BP3 (IMP3)	Fw: GTCAAGTGCAGAAGTTGTTGTC<br>Rv: GCAATCTGTCTTTGGTTTGGC
IL1A	Fw: GAATGACGCCCTCAATCAAAGT<br>Rv: TCATCTTGGGCAGTCACATACA
IL1B	Fw: AAACAGATGAAGTGCTCCTTCCAGG<br>Rv: TGGAGAACACCACTTGTTGCTCCA
IL1RI	Fw: GTGATTGTGAGCCCAGCTA<br>Rv: TGTTTGCAGGATTTTCCACA
IL1RA	Fw: GGCACTTGGAGACTTGTATGAAAGAT<br>Rv: TCGCTGAGTACCTGCCAAGA
IL6	Fw: GGCACTGGCAGAAAACAACC<br>Rv: GCAAGTCTCCTCATTGAATCC
IL8	Fw: CTGGCCGTGGCTCTCTTG<br>Rv: CCTTGGCAAAACTGCACCTT
CXCL12	Fw: GAACGCCAAGGTCGTGGTCGT<br>Rv: TCTGTAGCTCAGGCTGACGGGC
BNIP3	Fw: TGAGTCTGGACGGAGTAGCTC<br>Rv: CCCTGTTGGTATCTTGTGGTGT
BNIP3L	Fw: GTGGAAATGCACACCAGCAG<br>Rv: CTTGGGTGGAATGTTTTCGG
ICAM3	Fw: GTAACTGCCGCTCCGTTG<br>Rv: ACTTTGTCCCCGTCTTCGT
NEMO	Fw: GGTGGAGCACCTGAAGAGAT<br>Rv: CAGAGCCTGGCATTCCTTAG
CDK6	Fw: GTGCCCTGTCTCACCCATAC<br>Rv: GACCCATAAGCCACCAAGG
CASP8	Fw: CTGCTGGGGATGGCCACTGTG<br>Rv: TCGCCTCGAGGACATCGCTCTC
CASP10	Fw: TGCCAAGGTGAAGAGATACAGC<br>Rv: CCAGGGGCACAGGGAATACTA
WT1	Fw: GACCAGCTCAAAAGACACCAAAGGA<br>Rv: ACAGCTGAAGGGCTTTTCACTTGT
GATA4	Fw: AGAAAACGGAAGCCCAAGAACC<br>Rv: TGCTGTGCCCGTAGTGAGATGA
SOX17	Fw: CGCACGGAATTTGAACAGTA<br>Rv: GGATCAGGGACCTGTCACAC
TBX3	Fw: TGGGGACCTCTGATGAGTCCT<br>Rv: CCATGCTCCTCTTTGCTCTC
HoxA10	Fw: GCCCTTCCGAGAGCAGCAAAG<br>Rv: AGGTGGACGCTGCGGCTAATCTCTA
HoxD8	Fw: GATAACTTACAGAGACAGCCGATTTTTAC<br>Rv: CCAATATTACCACTGGACGATTTACA
P4HA1	Fw: CCCTGAGACTGGAAAATTGACCACAGC<br>Rv: GGGGTTCATACTGTCCTCCAACTCCA
ASPH	Fw: TTTCTCAAACTGCAGCCTCC<br>Rv: TGGGCACTTCCTACAGTTCC
ASPHD1	Fw: TCGAAGCTTTATGAGTGCCA<br>Rv: CAGCCAGGAGGGATCTTTAG
PTRF	Fw: GACTCAAAGCCAAAACTGCCC<br>Rv: CAAAAGCTGCTTTCACGTCCTT
GUSB	Fw: CAACGAGCCTGCGTCCCACC<br>Rv: ACGGAGCCCCCTTGTCTGCT
GAPDH	Fw: AACTGCTTGGCACCCCTGGC<br>Rv: CTGGAGAGCCCCTCGGCCAT
cMYC	Fw: CTTGTACCTGCAGGATCTGA<br>Rv: GTCGAGGAGAGCAGAGAATC
CDK6	Fw: GTGCCCTGTCTCACCCATAC<br>Rv: GACCCATAAGCCACCAAGG

HMGA2	Fw: AAGTTGTTCAGAAGAAGCCTGCTCA<br>Rv: TGGAAAGACCATGGCAATACAGAAT
PTPRF	Fw: ACCATGCTATGTGCCGCAG<br>Rv: CCTTGGTCGGATTCCTCACT
CD9	Fw: GAGGCACCAAGTGCATCAA<br>Rv: AGCCATAGTCCAATGGCAAG
NEMO	Fw: GGTGGAGCACCTGAAGAGAT<br>Rv: CAGAGCCTGGCATTCCTTAG
ICAM3	Fw: GTAACTGCCGCTCCGTTG<br>Rv: ACTTTGTCCCCGTCTTCGT
GATA2	Fw: AGCCGGCACCTGTTGTGCAA<br>Rv: TGACTTCTCCTGCATGCACT
OCT4	Fw: CCCCAGGGCCCCATTTTGGTACC<br>Rv: GGCACAAACTCCAGGTTTTC
MDB3	Fw: GGTGACCAAGATTACCAACCAC<br>Rv: GCTCCTCAGAATGTCGAAGG
DIDO3	Fw: ACTCTTGCCCTTTGAGGGACCA<br>Rv: TCGGGTCCGCTTTTCGTCCA

Table 2: List of all primary and secondary antibodies used in the study.

Primary antibodies	*Reference*	*Commercial provider*
Anti-human IGF2R	AF2447	R&D Systems, Minneapolis, MN
Anti-human ASPHD1	Ab19731	Abcam, Cambridge, MA
Anti-human P4HA1	HPA026593	Sigma-Aldrich (St Louis,. MO).
Anti-human IL1A/IL1-1F1	MAB200	R&D Systems, Minneapolis, MN
Anti-human IL1B	sc-7884	Santa Cruz Biotechnology, Inc
Anti-human IL1RI	AF269	R&D Systems, Minneapolis, MN
Anti-human IGF2BP2 (IMP2)	ab128175	Abcam, Cambridge, MA
Anti-human IGF2BP3 (IMP3)	ab177942	Abcam, Cambridge, MA
Anti-hnRNP U antibody	ab10297	Abcam, Cambridge, MA
Anti-α-tubulin	CP06	Calbiochem, La Jolla, CA, USA
Anti-GAPDH antibody	Ab9485	Abcam, Cambridge, MA

Secondary antibodies	*Reference*	*Commercial provider*
Goat Anti-rabbit HRP	P0448	Dako, Glostrup Denmark
Rabbit Anti-Mouse-HRP	P0260	Dako, Glostrup Denmark
Rabbit Anti-Goat HRP	P0449	Dako, Glostrup Denmark
Goat anti-Rabbit IgG (H+L) Secondary Antibody, Alexa Fluor® 546 conjugate	A-11035	Invitrogen, Carlsbad, CA, USA
Goat anti-Mouse IgG (H+L) Secondary Antibody, Alexa Fluor® 546 conjugate	A-11030	Invitrogen, Carlsbad, CA, USA
Donkey anti-Goat IgG (H+L) Secondary Antibody, Alexa Fluor® 546 conjugate	A-11056	Invitrogen, Carlsbad, CA, USA

DAPI	32670	Sigma-Aldrich (St Louis,. MO).

a

Identification (IPA) ; nuclear and cytosol fractions from hCPC and hMSC

IPA location	hCPC Cytopl	hCPC Nucl	SubT	MSC Cytopl	MSC Nucl	SubT	Total
Cytoplasm	748	523	1271	707	469	1176	2447
Extracellular Space	44	47	91	28	40	68	159
Nucleus	223	369	592	166	348	514	1106
Plasma Membrane	84	103	187	70	109	179	366
unknown	91	60	151	98	73	171	322
Total general	**1190**	**1102**	**2292**	**1069**	**1039**	**2108**	**4400**

b

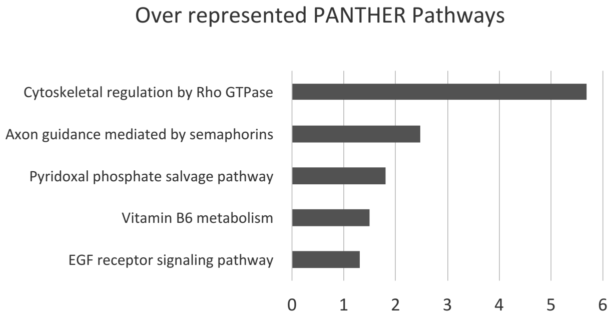

c

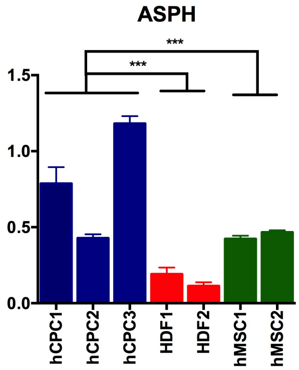

d

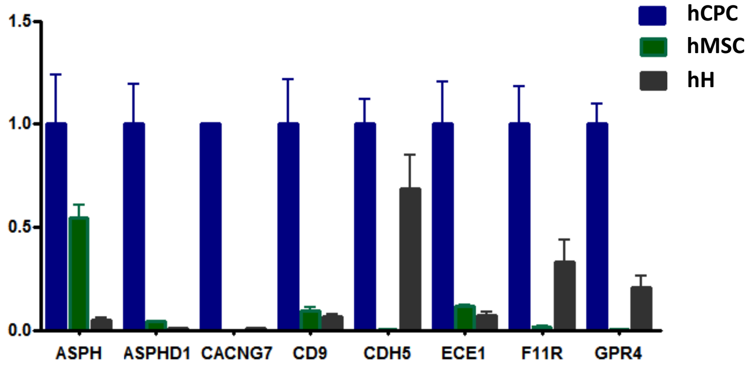

Validation of cytoplasmic overexpressed proteins in hCPC. a) Protein identification (IPA) in subcellular fractions prepared from hCPC (hCPC3) and hMSC (hMSC19**); b)** PANTHER Pathway analysis of cytoplasmic overexpressed proteins in hCPC; **c)** Comparative RT-qPCR expression analysis of *ASPH* in the three independent isolates of hCPC (hCPC 1–3), two human fibroblasts (HDF1 and F3) and two hMSC isolates (MSC19 and MSC45). Assays were performed three times and data are expressed as mean ± SD; black lines summarize p-values (***<0.002) for hCPC *vs*. fibroblasts or hMSC (one-way ANOVA analysis of variance followed by the Bonferroni correction for multiple comparison); **d)** RT-qPCR comparative analysis of some of the identified cytoplasmic overexpressed proteins in hCPC, with other functions previously described as highly differentially overexpressed by hCPC. Expression in hCPC (hCPC1) was compared with hMSC (hMSC19) and whole human heart (hH) tissue; relative expression values to hCPC.

Supplementary Fig. S2

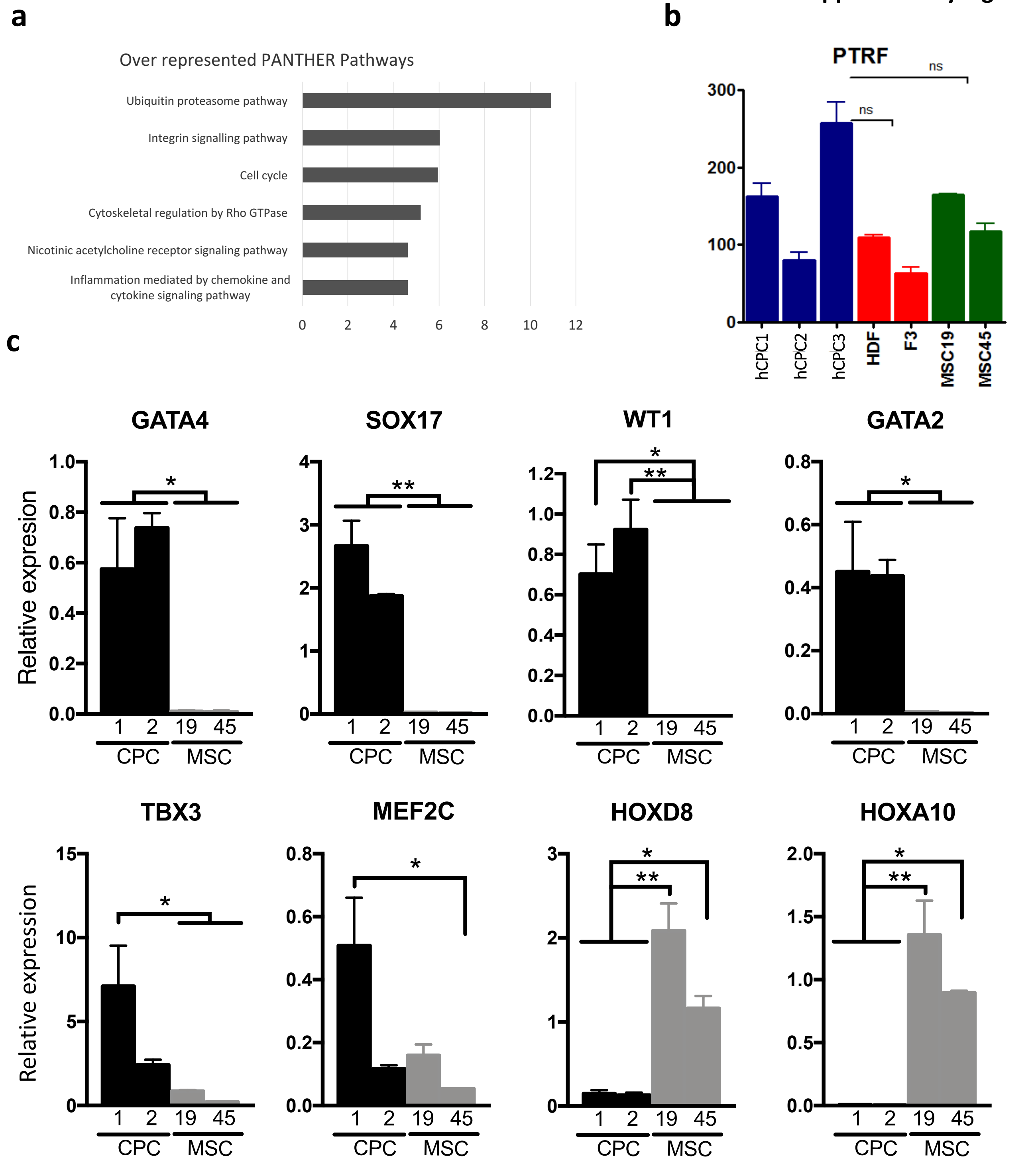

Validation of nuclear overexpressed proteins in hCPC. a) PANTHER Pathway analysis of nuclear overexpressed proteins in hCPC. **b)** Comparative RT-qPCR expression analysis of *PTRF* in the three independent isolates of hCPC (hCPC 1–3), two human fibroblasts (HDF1 and F3) and two hMSC isolates (MSC19 and MSC45). Assays were performed three times, normalized against *GAPDH* and data expressed as mean ± SD; black lines summarize p-values (one-way ANOVA analysis of variance followed by the Bonferroni correction for multiple comparison; ns, non-significant. **c)** RT-qPCR confirmation of some transcriptional factors defined by RNAseq (Toran et al., 2019) as significantly overexpressed (*GATA4*, *SOX17*, *WT1*, *GATA2*, *TBX3* and *MEF*) or downregulated (*HOXD8* and *HOXA10*) in hCPC *vs.* hMSC. Two hCPC isolates (1 and 2) were compared with two hMSC isolates (MSC19 and MSC45). Assays were performed three times and data are expressed as mean ± SD; black lines summarize p-values (**<0.02; *<0.05; one-way ANOVA analysis of variance followed by the Bonferroni correction for multiple comparison).

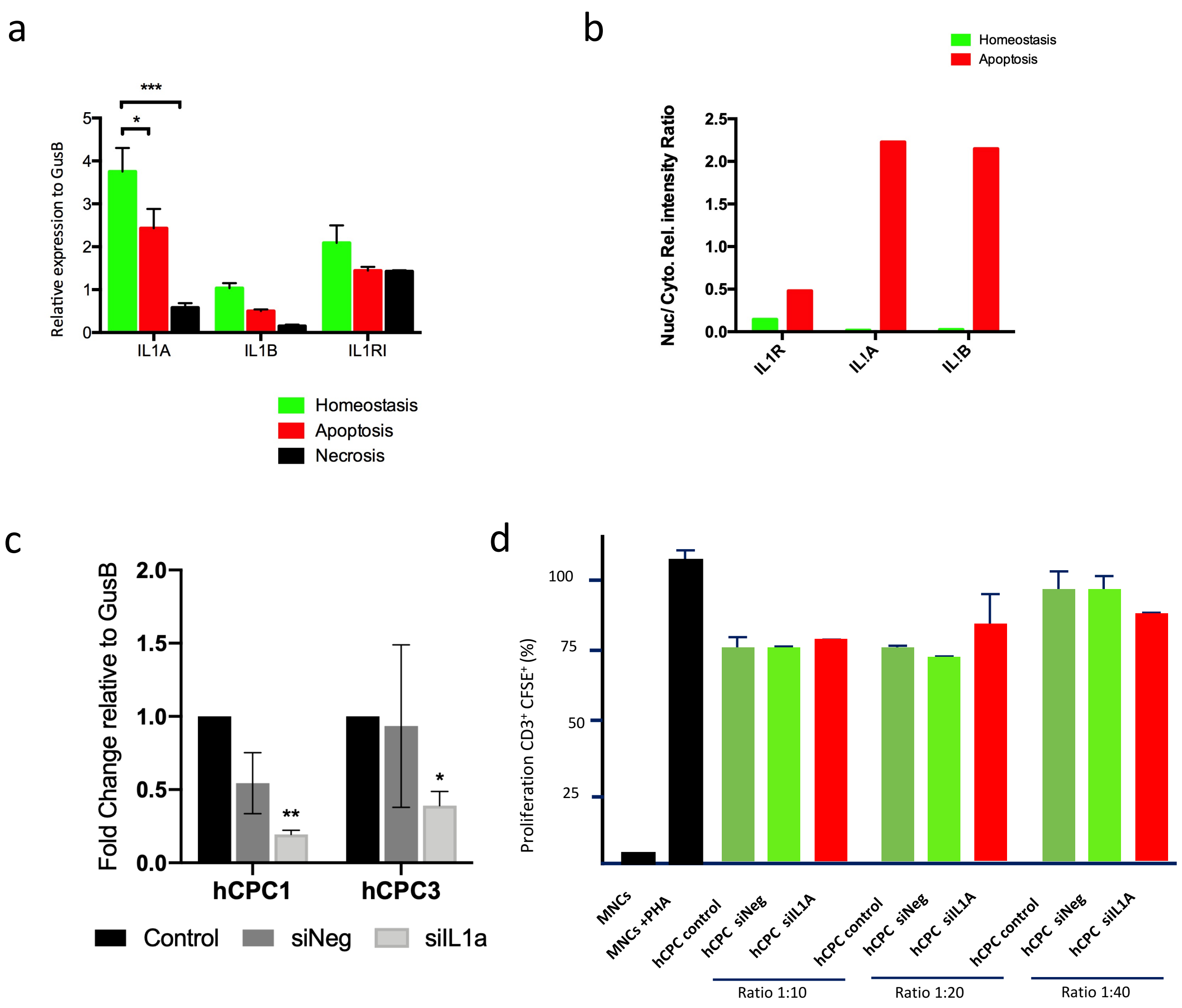

IL1A response to apoptosis/necrosis upon oxidative damage and evaluation of a potential role in immunoregulation. a) Comparative evaluation of IL1A, IL1B and IL1R1 expression by RT-qPCR in hCPC1, in homeostasis, and upon induction of apoptosis or necrosis, and relative to GusB **b)** Densitometric analysis of the representative western blot shown in Figure 3d; nuclear/cytoplasmic ratio for IL1R, IL1A and IL1B are compared in in hCPC3 in homeostasis of after apoptosis induction. **c, d)** Evaluation of the potential role of IL1A in hCPC immunoregulation capacity. **c)** Confirmation of downregulation of IL1A (>70 %) in hCPC (1,3) transfected with sIIL1A (10 nM) compared with a negative control (siNeg) and untransfected control cells (control), by RT-qPCR relative to the expression of GusB; Assays were performed three times and data expressed as mean ± SD of the results relative to GusB; black lines summarize p-values (**<0.02; *<0.05; one-way ANOVA analysis of variance followed by the Bonferroni correction for multiple comparison). **d)** Phytohemagglutinin-stimulated human CD3 T cells, labeled with CFSE, were co-cultivated with native hCPC cells (hCPC3), CPC-IL1A downregulated cells (hCPC siIL1A) or negative control transfected cells (hCPC siNeg).All three samples (hCPC, hCPC siIL1A and hCPC siNeg) demonstrated similar immunoregulatory capacity (% of proliferating CD3+ cells) at the higher cell doses analyzed (1:10–1:20);

a

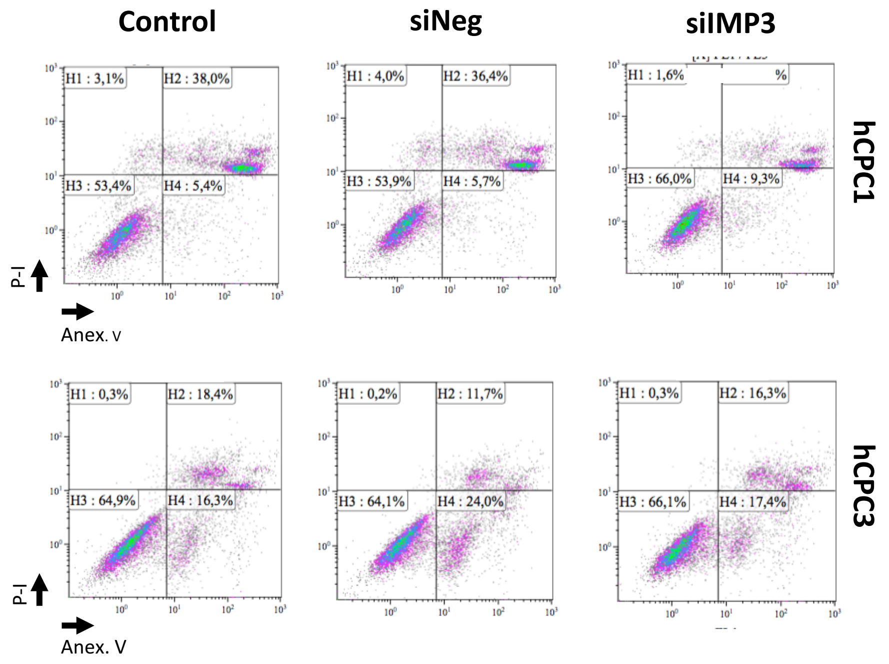

b

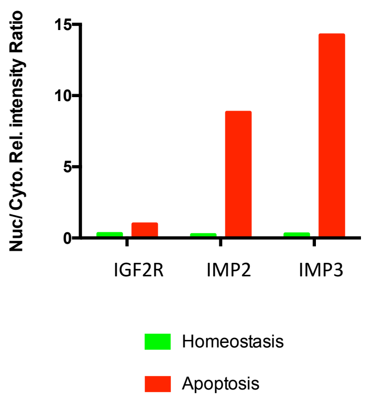

c

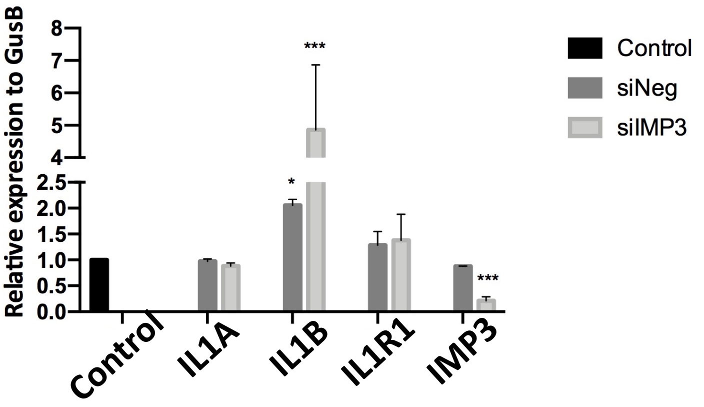

Evaluation of IMP3 and IL1A functional interaction and response to apoptosis/necrosis upon oxidative damage. a) Analysis of the effects of IMP3 downregulation on hCPC1 or hCPC3 response to oxidative damage induced by H_2O_2. hCPC control, siIMP3- or siNeg-transfected cells were exposed to H_2O_2 (500 μM) during 48 h; cultures were stained with the AnnexinV/ Propidium iodide (Anex.V / PI) and homeostatic viable (H3: Anex.V-/ PI-), apoptotic (H4: Anex.V+/ PI-), late apoptotic (H2: Anex.V+/ PI+) or necrotic (H1: Anex.V-/ PI+) cells were quantified by cytometry. Data correspond to a representative experiment; assays were performed three times and data expressed as mean ± SD are included in Figure 4E; **b)** Densitometric analysis of the representative western blot shown in Figure 5b; nuclear/cytoplasmic ratio for IGF2R, IMP2 and IMP3, in hCPC3 are compared in homeostasis of after apoptosis of induction. **C)** Evaluation of potential role of IMP3 in regulation of IL1A expression in hCPC. Using the hCPC3 isolate, hCPC control, siIMP3- or siNeg-transfected cells were evaluated, 48 h after transfection, by RT-qPCR. Assays were performed three times and data expressed as mean ± SD of the results relative to GusB; black lines summarize p-values (**<0.02; *<0.05; one-way ANOVA analysis of variance followed by the Bonferroni correction for multiple comparison).

Supplementary Fig. S5 online

full-length blots/gels

Figure 1f

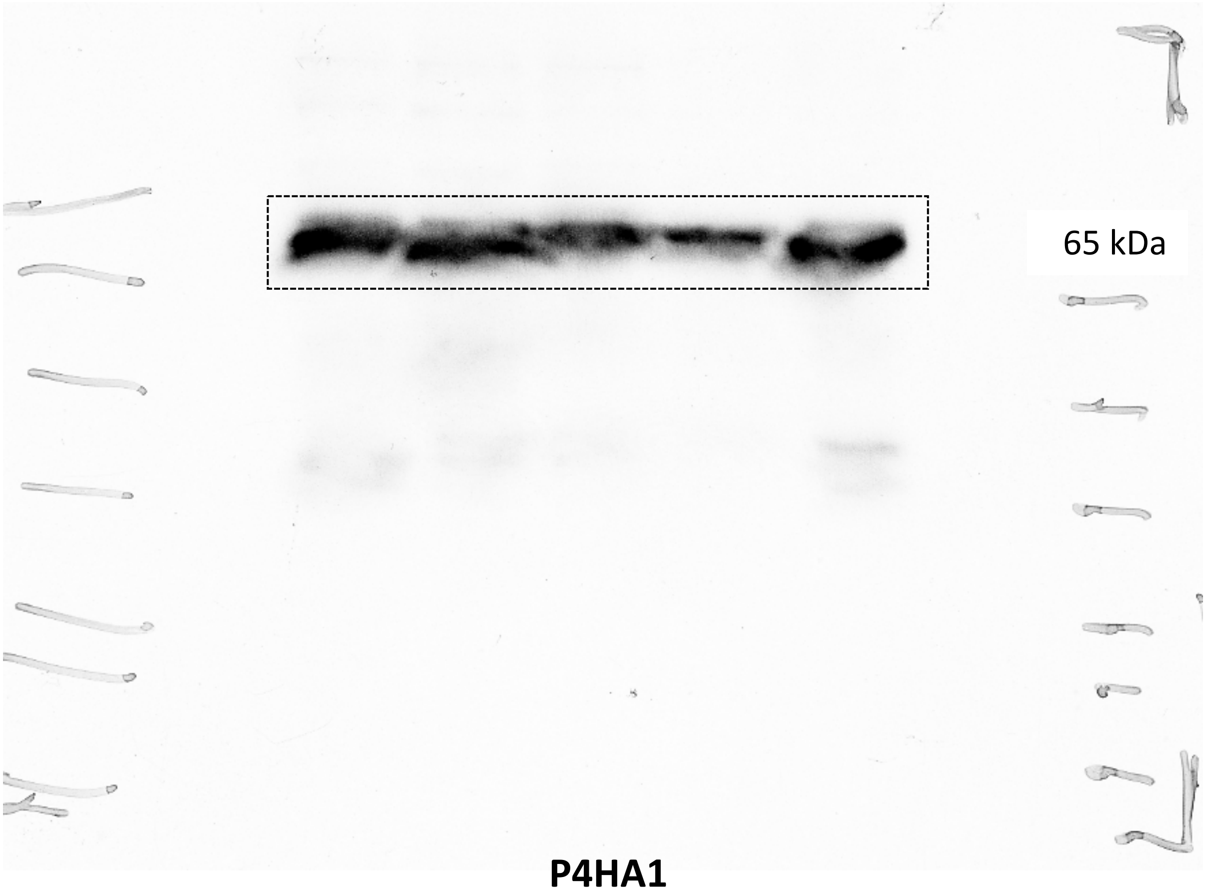

P4HA1

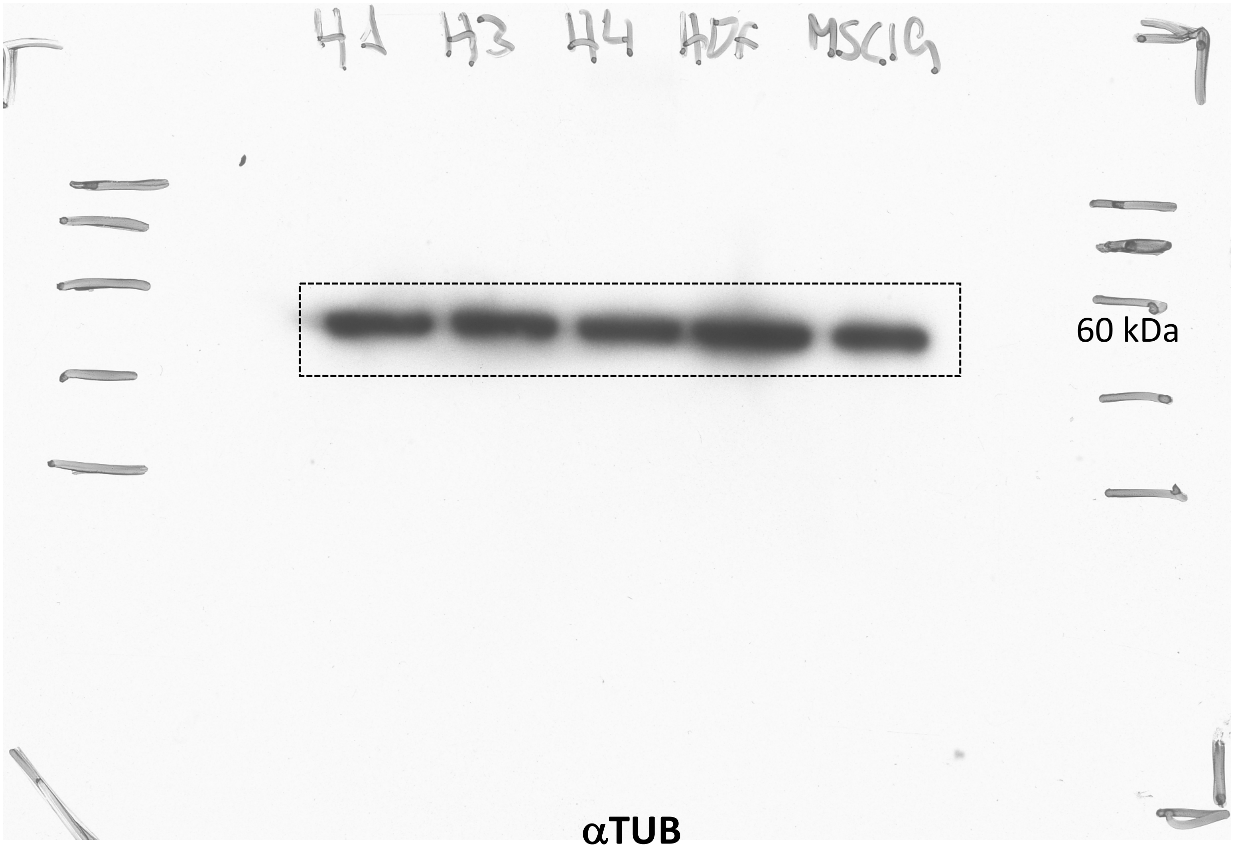

αTUB

Figure 2f

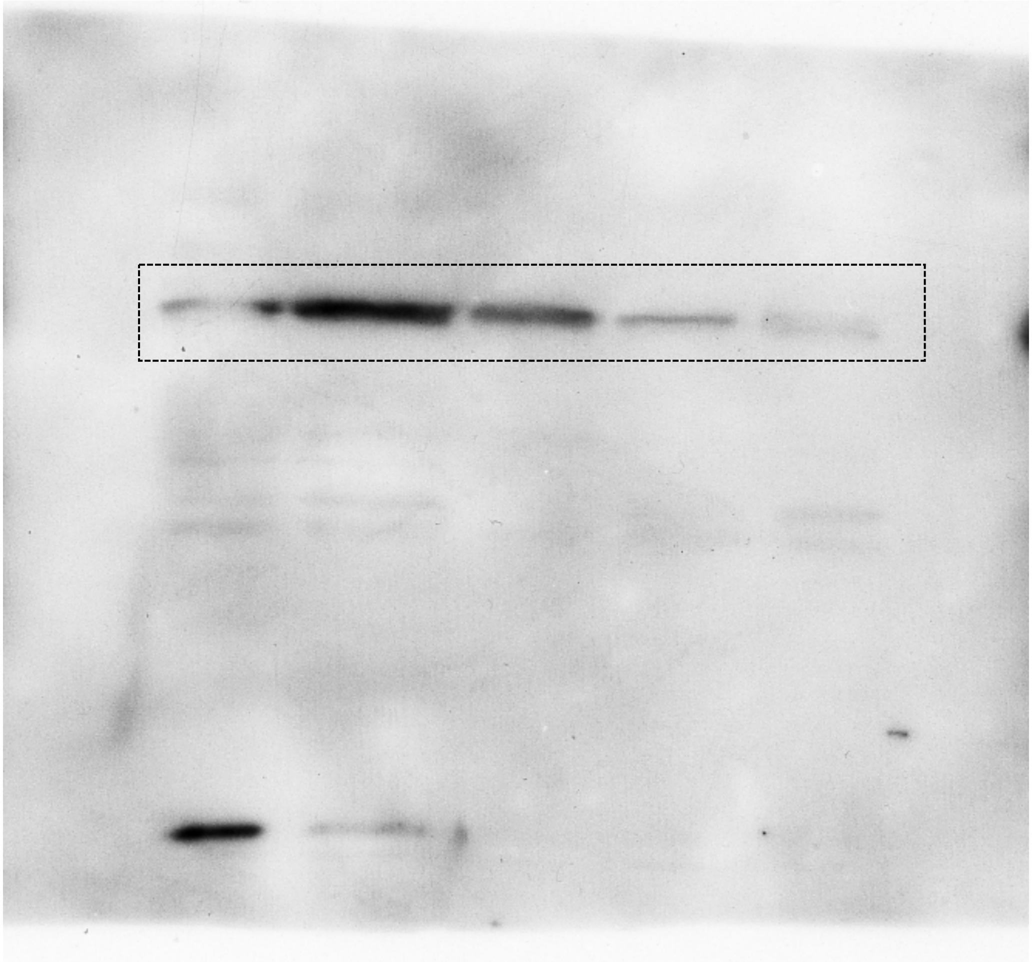

ASPHD1

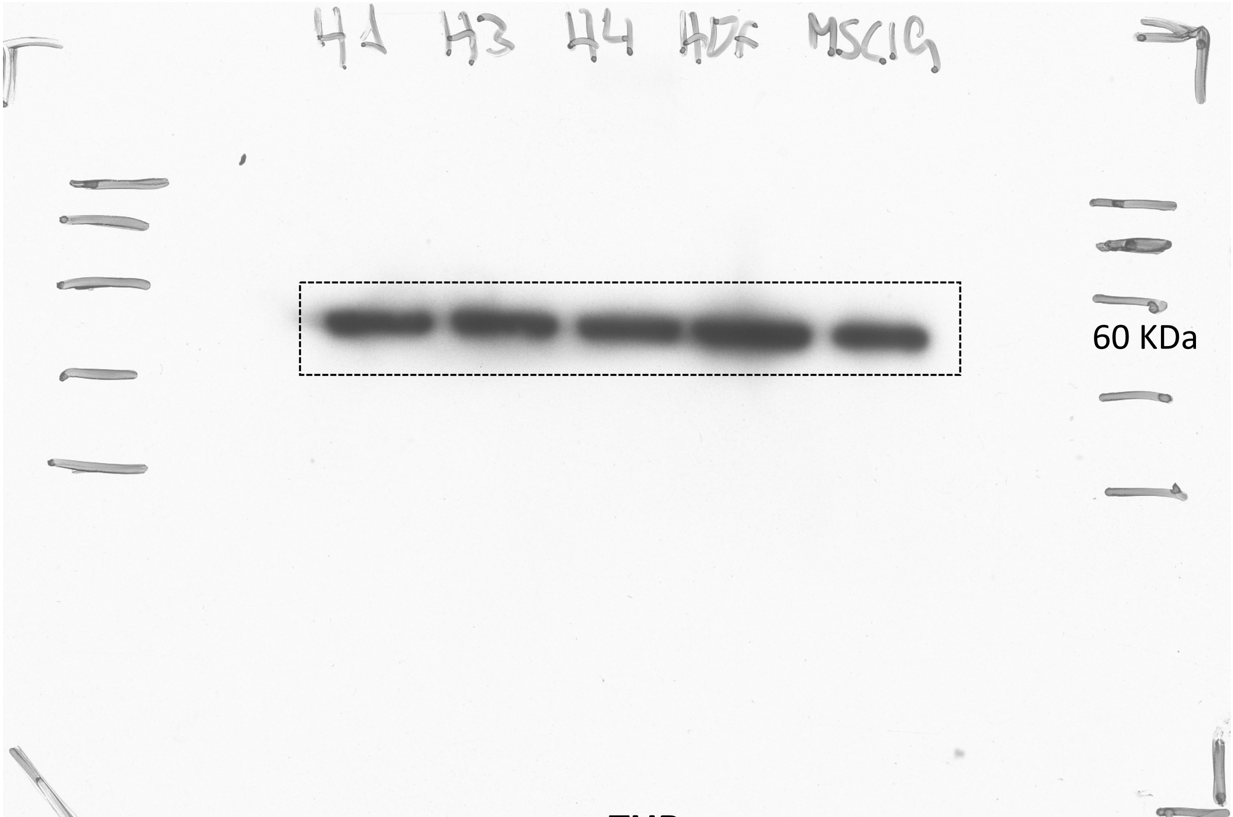

αTUB

Note: same membrane used in Fig 1f, reused for anti-ASPHD1

Fig. 3e (Homeostasis)

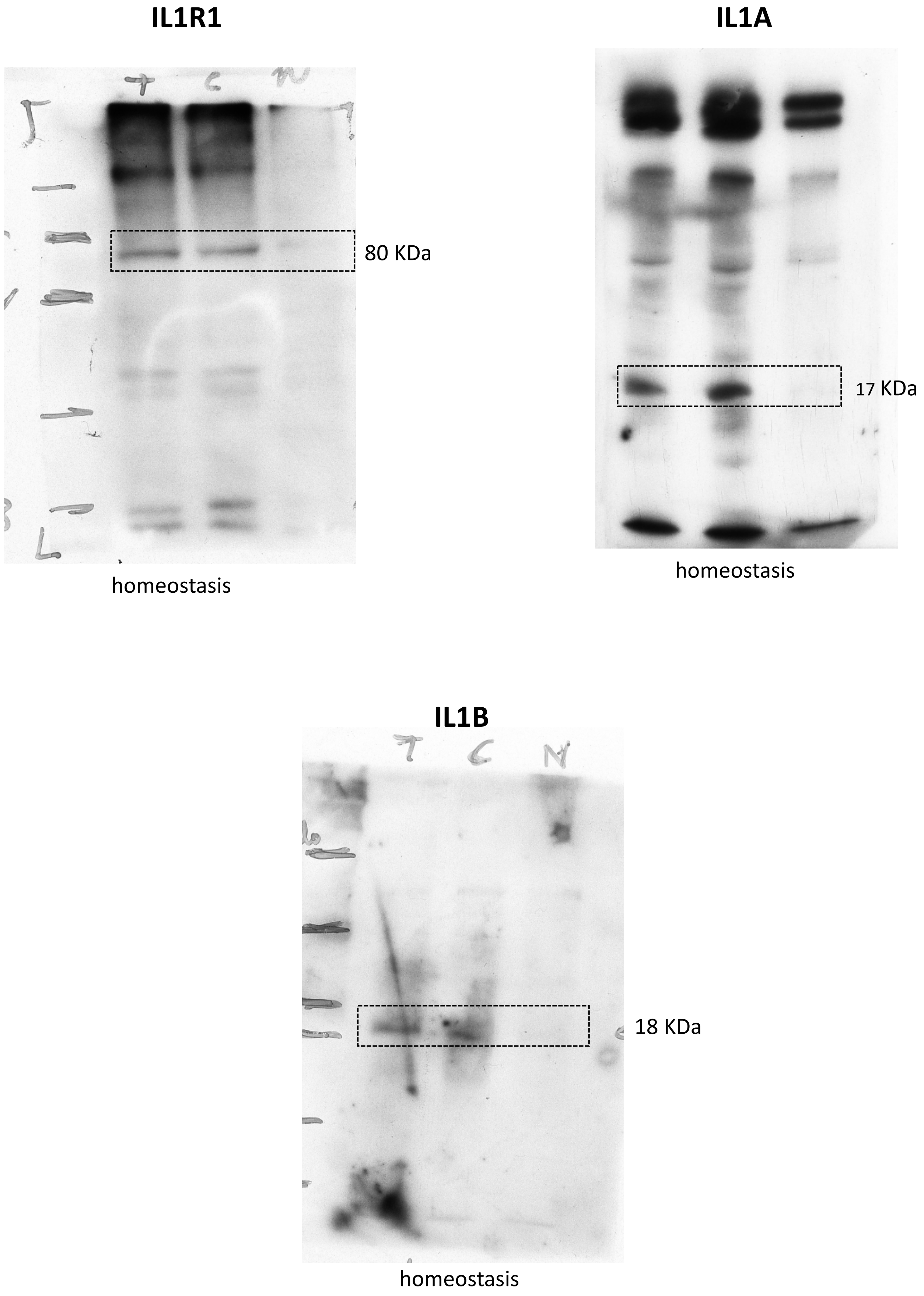

Fig. 3e (Homeostasis)

hnRNPU

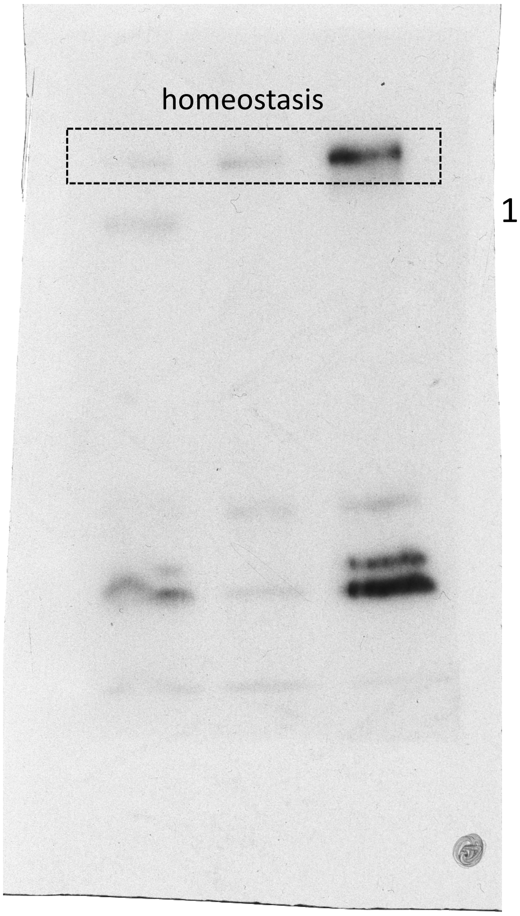

120 KDa

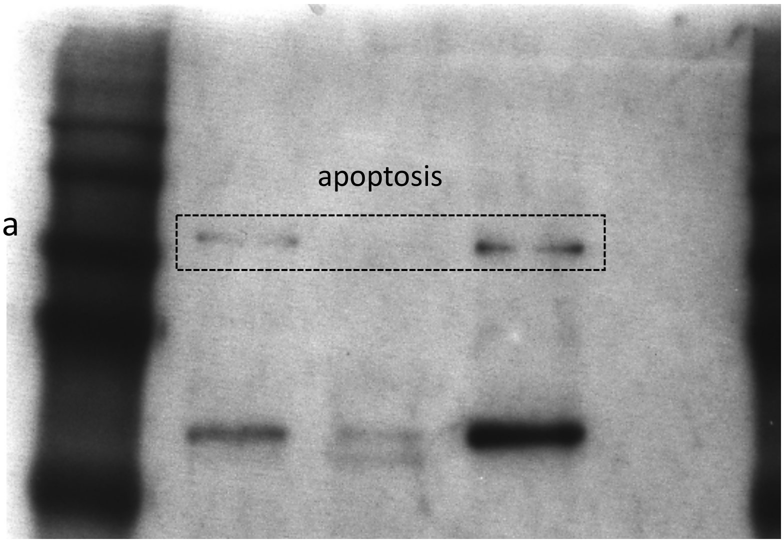

GAPDH

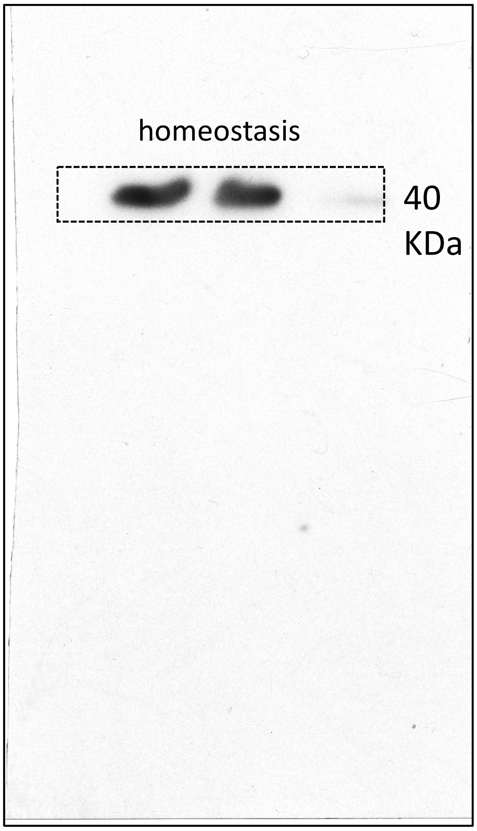

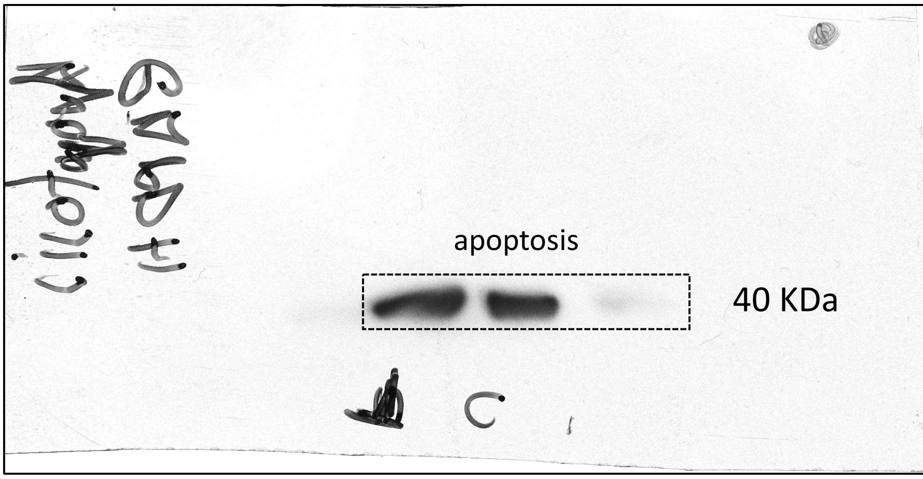

Note: the same membrane used in Fig 5b was reused for these analysis. So the controls for hnRNPU and GAPDH are the same.

Figure 3e (apoptosis)

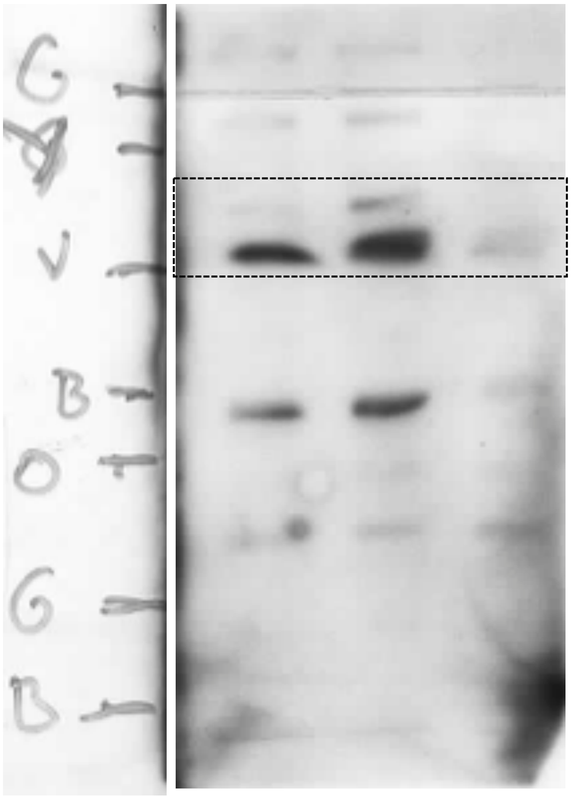

IL1R1
apoptosis

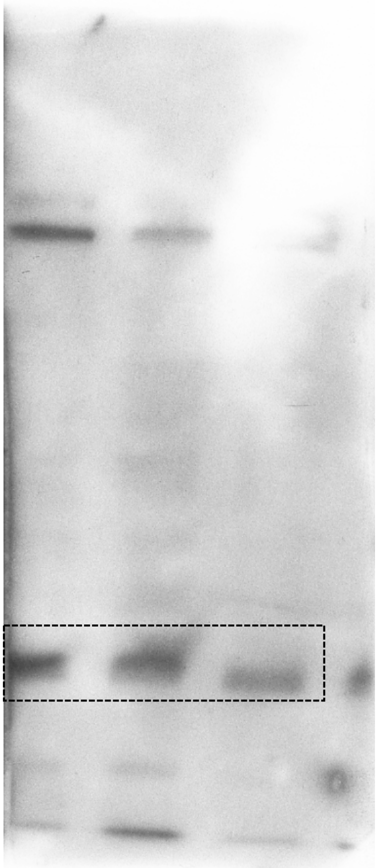

IL1A
apoptosis

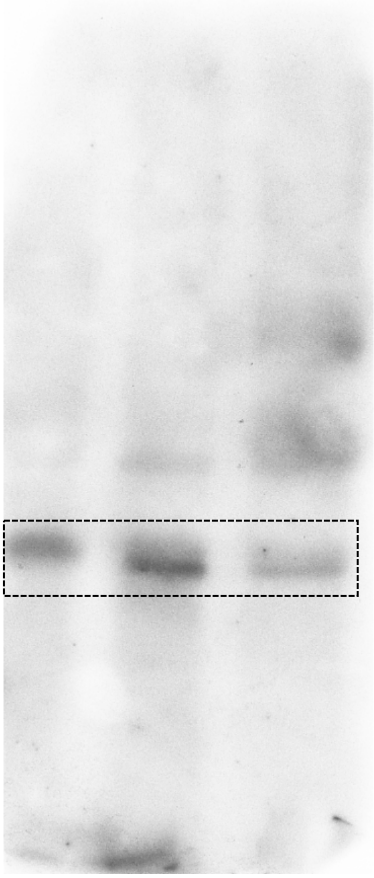

IL1B
apoptosis

Figure 3g bis

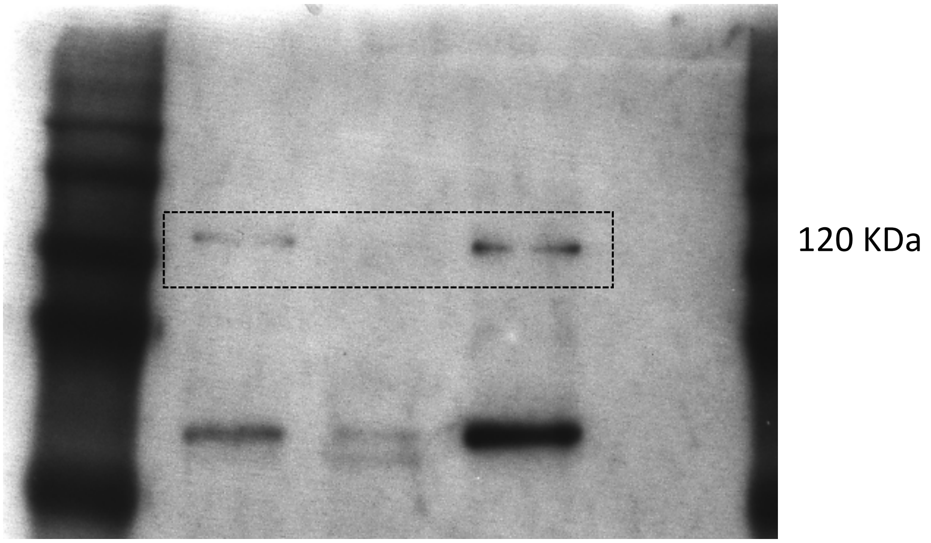

hnRNPU

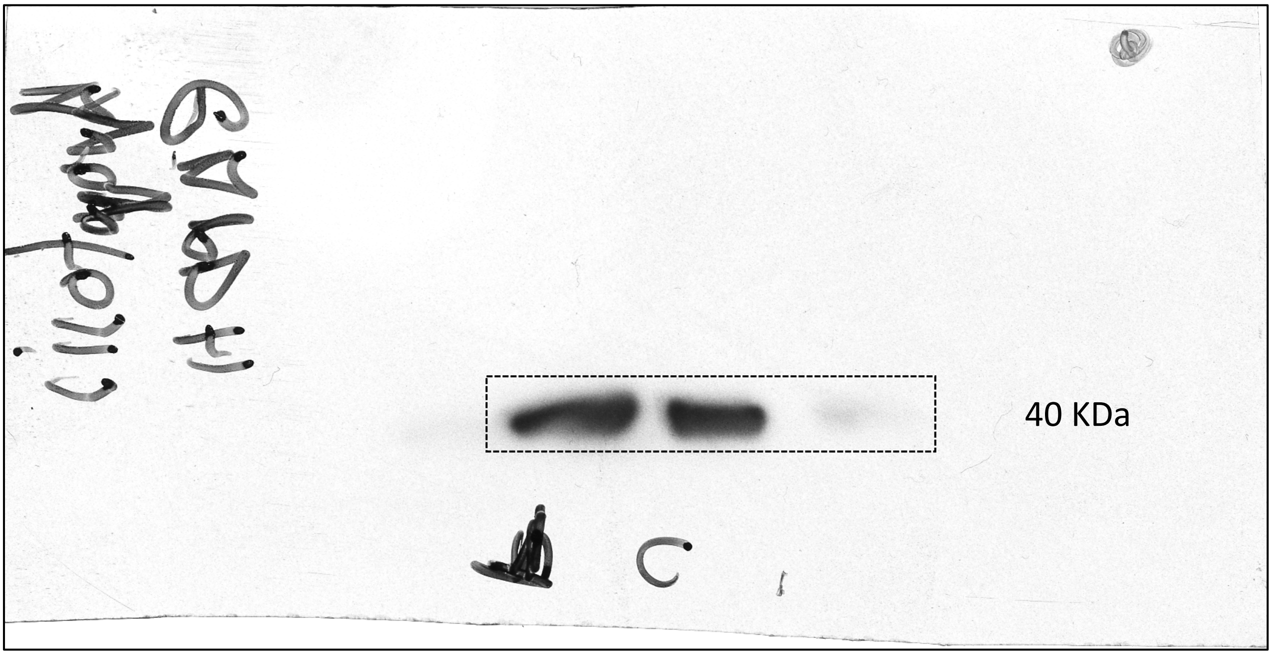

GAPDH

Figure 4c

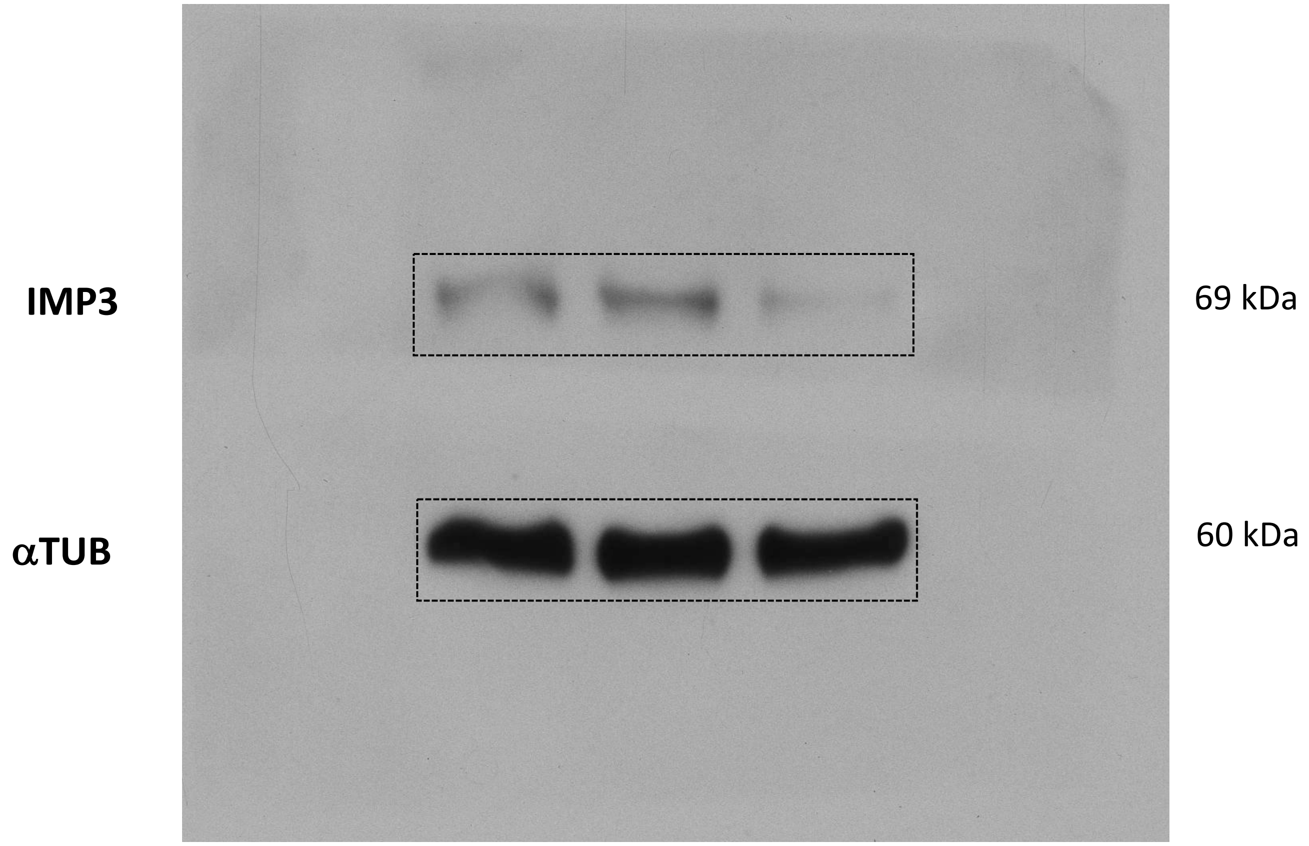

Figure 5b

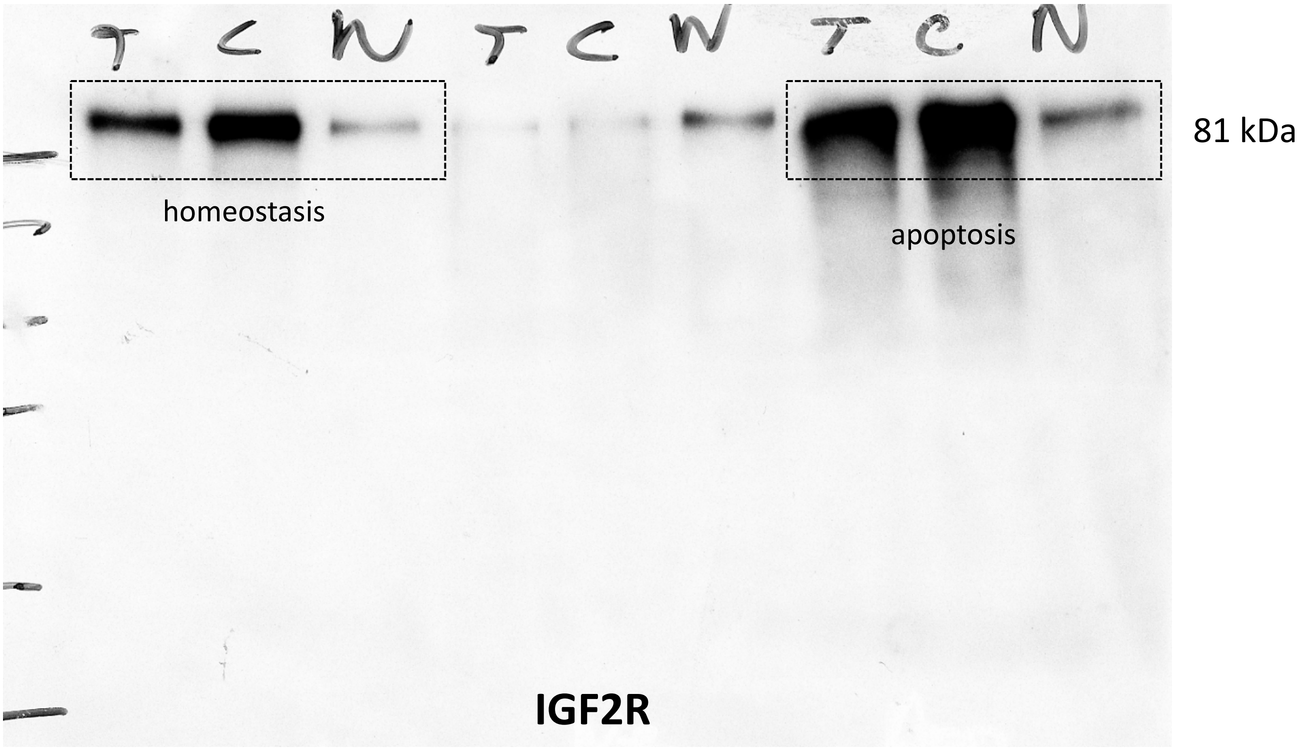

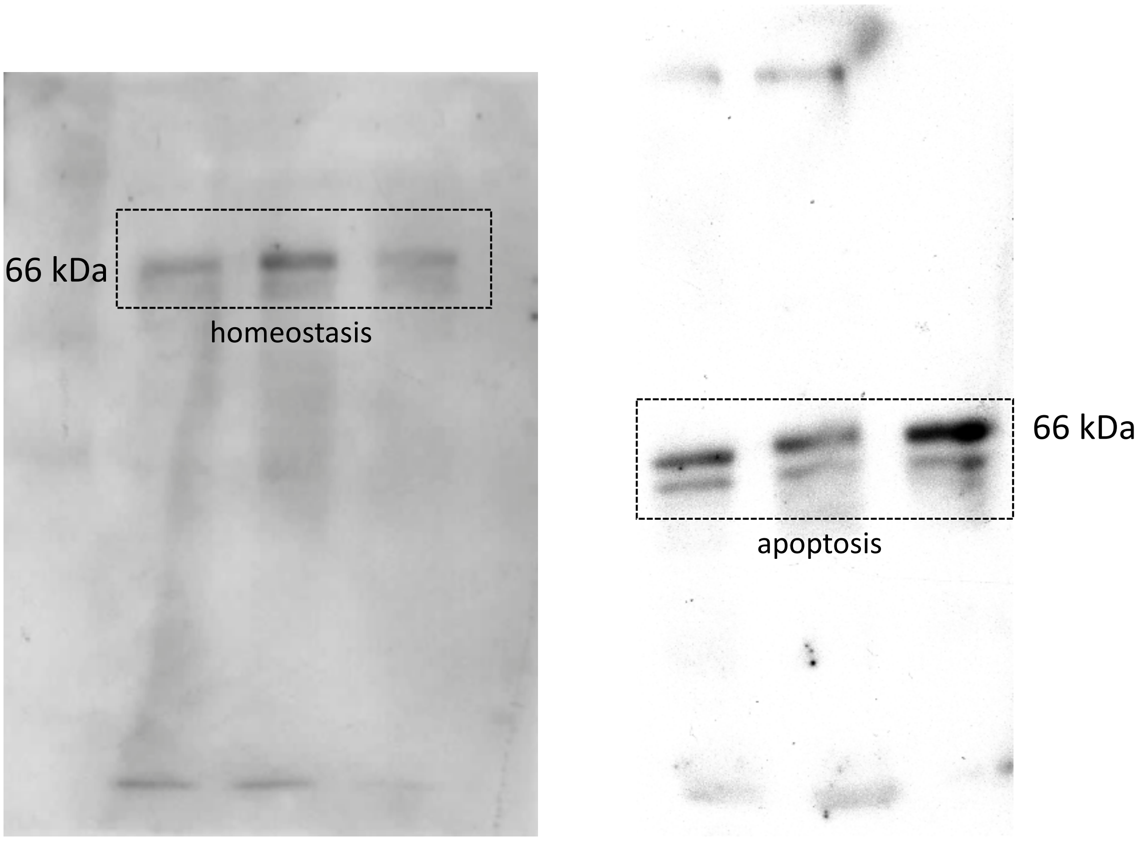

IMP2

Figure 5b bis

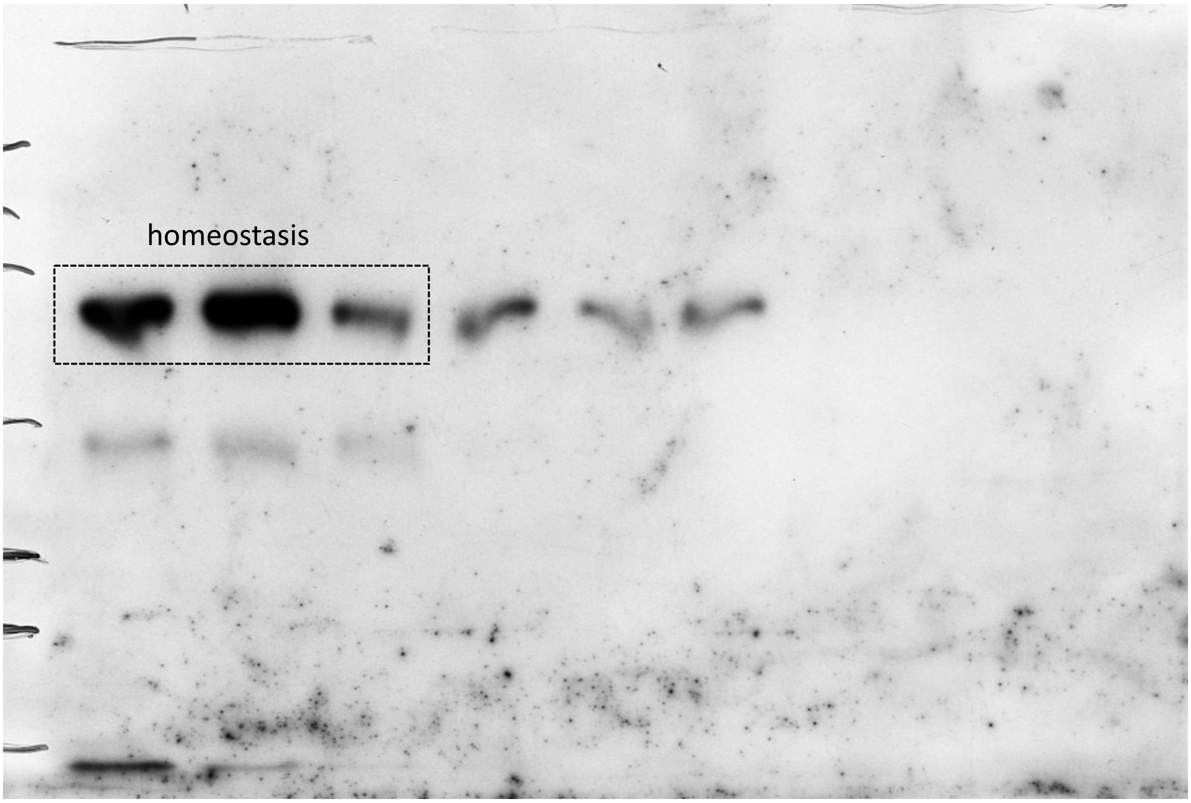

69 kDa

IMP3

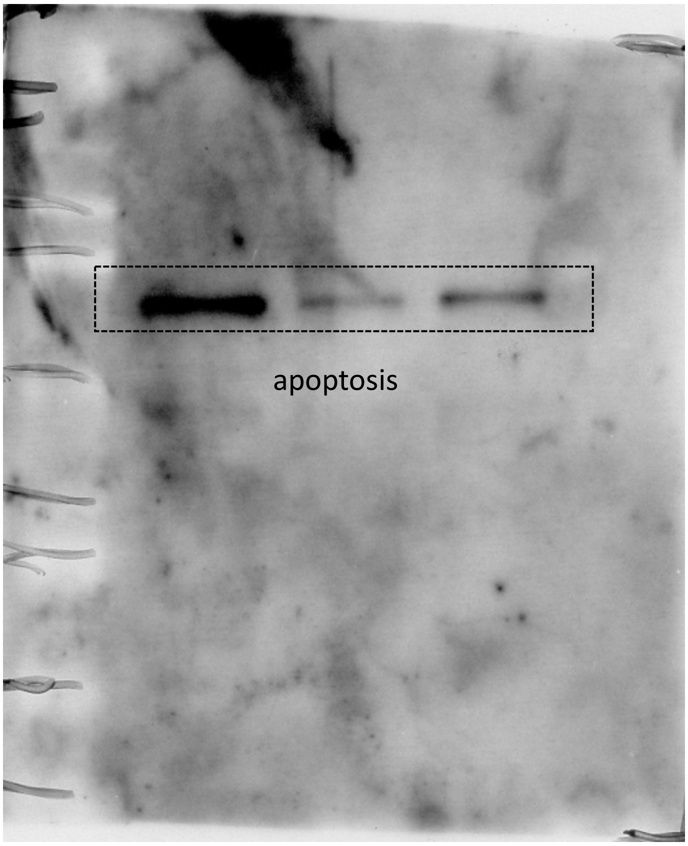

69 kDa

Figure 5b bis

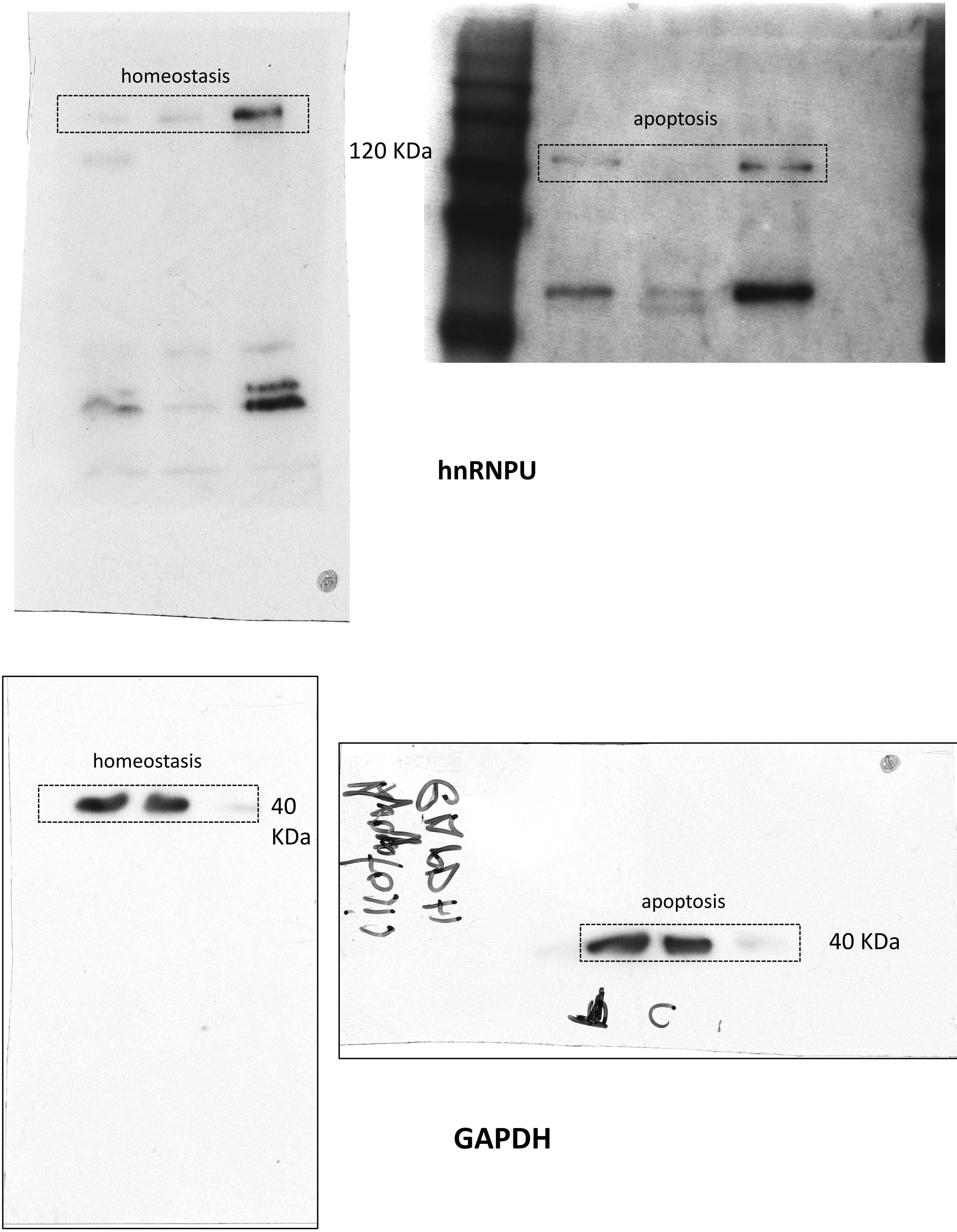

Supplement: Supplementary file 1 — Supplementary Information. [file 41598_2021_3956_MOESM1_ESM.pdf]
